# Active salt transport and countercurrent exchange as the basis of urine concentration: a proposal grounded on the microanatomy of the renal medulla

**DOI:** 10.1007/s00424-026-03152-5

**Published:** 2026-02-14

**Authors:** Wilhelm Kriz, Brigitte Kaissling, Michel Le Hir

**Affiliations:** 1 Department of Neuroanatomy, Medical Faculty Mannheim, University of Heidelberg, Ludolf-Krehl-Str. 13-17, Tridomus C, Mannheim, 68167 Germany; 2 Institute of Anatomy, University of Zurich, Zurich, Switzerland

**Keywords:** Urine concentrating mechanism, Countercurrent exchange, Salt reabsorption, Salt secretion, Urea recycling

## Abstract

The longitudinal arrangement of tubules and vessels in the renal medulla has in the early fifties of the last century given birth to the concept of countercurrent multiplication (CCM). Since then, enormous research has been expended, but a consent on a common concept of the urinary concentrating mechanism has not been reached. Strictly based on the microanatomy of the renal medulla and the transport capacities of the involved tubules and vessels, we propose a mechanism, in which countercurrent exchange (CCE) operates at different levels in the outer and inner medulla to create gradients of salt and urea and to impede their dissipation. Salt release by ascending thick and thin loop limbs (TALs and ATLs) including the bend segments and urea release by terminal collecting ducts (CDs) increase at any medullary level solute concentrations in ascending vasa recta (AVRs) above those in descending loop limbs (DLs) and CDs leading to water extraction from both resulting in final urine concentration. Solute loss by washout is minimized by counter-current exchange between AVRs and descending vasa recta (DVRs), continuously balanced through addition of salt by ATLs and urea by terminal CDs.

## Introduction

During phylogenesis in mammals and birds a renal medulla has developed in response to the necessity to conserve water by excreting a concentrated urine. Loops of Henle, collecting ducts, and a specific blood supply through vascular bundles have been combined into a complex structural system that accounts for this function. In this system the counter-current arrangement of the descending and ascending limbs of Henle’s loops as well as of the descending and ascending vasa recta within the vascular bundles is eye-catching and has prompted researchers to consider the counter-current organization of the medulla as essential feature underlying the urine concentrating function. Two mechanisms have been considered: countercurrent exchange (CCE) and countercurrent multiplication (CCM).

The CCM-hypothesis, was first published in two papers in the early fifties of the past century by Wirz, Hargitay and Kuhn [36, 123], and republished in 2001 [35]. Its schematic representation is found in most publications on renal physiology for students and medical doctors. It relies on an active transport of some substance (in the present context: salt) from the ascending limb along its entire length into the descending limb creating a longitudinal salt gradient with the highest salt concentration at the loop bend. At any level of the loop the up-hill transport from the ascending limb into the descending limb, the so-called “single concentrating effect”, has to overcome just a small gradient. This mechanism was first experimentally established in artificial tubes [123] and has been subsequently applied to the renal medulla, albeit with significant deviations from the original conditions [122].

From a structural point of view, the prerequisites for CCM are not apparent in the medulla. At no place in the outer medulla (OM) is the majority of descending thin limbs (DTLs) juxtaposed to thick ascending limbs (TALs), furthermore their functional abilities gradually change along the descent. Moreover, the central idea of CCM that at any level of the OM a small up-hill transport of sodium by the TALs, the “single concentrating effect”, is multiplied by flow through the loop has never been documented. In contrast, TALs use at any level of the OM its full capacity to pump salt into the interstitial space [17, 37]. In addition, the dense arrangement of the AVRs at the entry into the medulla is hardly compatible with a CCM mechanism.

With respect to the inner medulla (IM) a CCM mechanism has never been seriously proposed.

CCE as a pure passive process can indeed delay the depletion of a given solute gradient but cannot maintain the gradient. However, if the solute losses along with solute outflow or dilution by water uptake are permanently balanced by solute addition from an external source, a solute gradient can well be established and maintained. Precisely this mechanism stabilizes the solute gradients in the medulla.

It is widely accepted that salt and urea represent the essential constituents of the osmotic gradient [104, 118], which drives water reabsorption from CDs in antidiuresis. The contributions of other solutes are of minor relevance. Continuous reabsorption of salt by the TALs, secretion of salt into the long descending limbs (LDTLs) in the inner stripe (IS) and the recycling of urea, as basis for release of urea from the terminal CDs, ensure the build-up of the salt and urea gradients in OM and IM.

We propose that the maintenance of the salt and urea gradients in the medulla during antidiuresis is based on CCE alone. The aim of this proposal is to establish a basis for further discussions and mathematical modelling of the urine concentrating mechanism without CCM.

## The structure of the mammalian renal medulla

Among species the basic common structure of the renal medulla reveals many variations that generally correlate with differences in the urine concentrating ability. This opens the chance of well-grounded structure-function correlations concerning various aspects of the concentrating mechanism.

Most structural, immune-cytochemical and physiological data are available from the rat and to a minor degree from the mouse, both species with a high urine concentrating ability. Humans and the rabbit have a much lower concentrating ability, their renal medulla is differently structured in some features. However, the concentrating mechanism that we describe for the rat is adaptable to the renal medulla in humans and other low concentrators. This will be separately discussed at the end.

## The structural organisation of the rat renal medulla

The renal medulla is made up of the loops of Henle, the medullary collecting ducts and a specific vascular system differently arranged in the three medullary regions, the outer stripe (OS), the inner stripe (IS), which together make up the outer medulla (OM) and the inner medulla (IM) [45, 56].

The rat kidney consists of roughly 30.000 nephrons of which about two thirds are equipped with short loops and one third with long loops of Henle [56, 111].

**Fig. 1:**
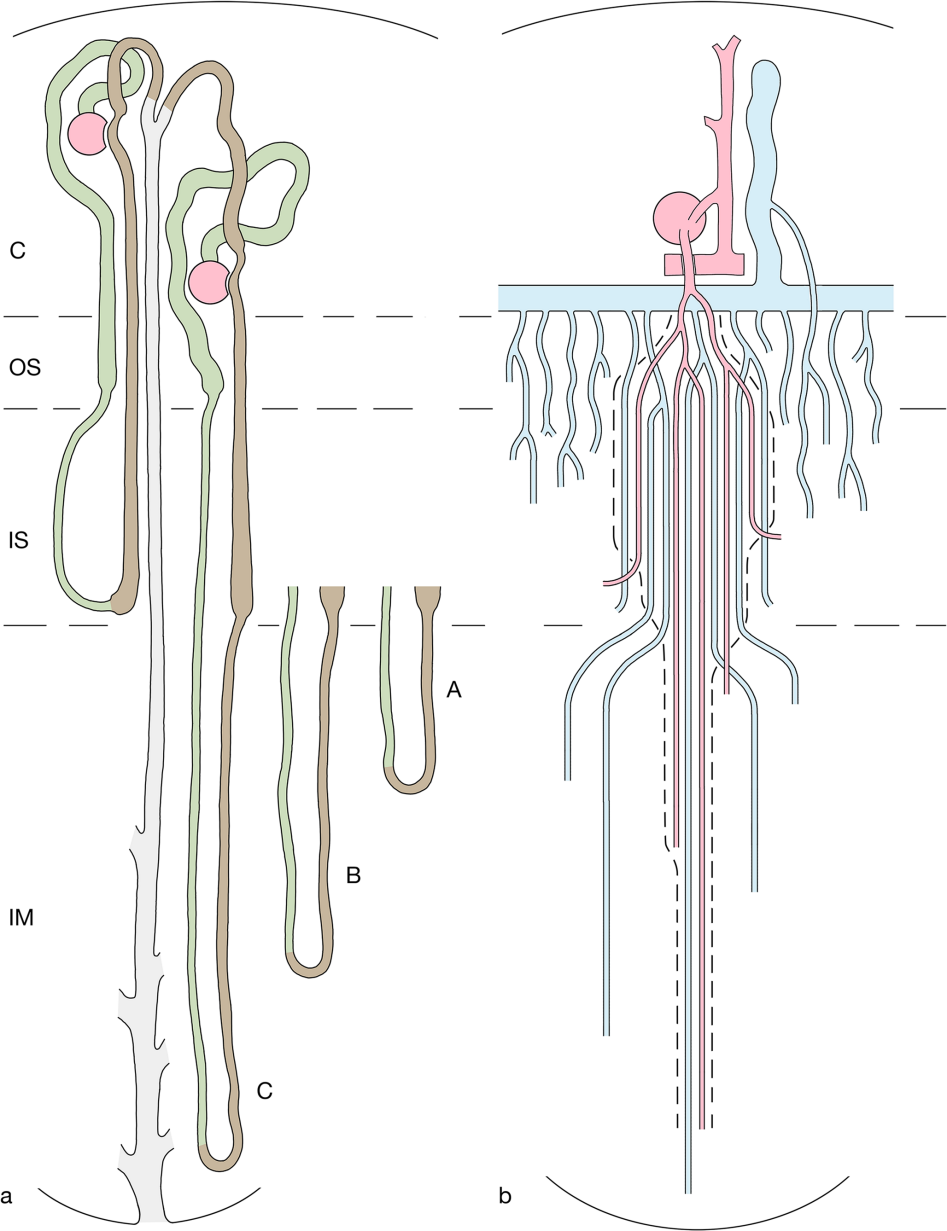
Schematics showing the relevant components of the rat kidney. presented in longitudinal sections through the cortex (C), outer stripe (OS), inner stripe (IS) and inner medulla (IM). a Nephrons and collecting ducts A short-looped nephron starting in the mid-cortex, a long-looped nephron starting in the juxtamedullary region and a collecting duct are shown. Proximal tubules and descending limbs of Henle’s loop are shown in green, ascending limbs and distal tubules are shown in brown, collecting ducts in pale grey. Since the long loops of Henle extend to different levels in the IM, for simplicity we consider three types: group A short long loops, group B long loops of intermediate length, group C long long loops. b Medullary vasculature The medullary perfusion starts with an efferent arteriole (EA) of a juxtamedullary glomerulus, splitting within the OS into descending vasa recta (DVRs, red), which establish together with ascending vasa recta (AVRs, blue) the vascular bundles (VB). DVRs traverse the IS within the VBs, a fraction of them leaves the bundle at intermediate and deep levels (only two are shown) of the IS to supply the tubules in the inter-bundle region (IBR outside the hatched line). The remainder of DVRs descend into the IM (three are shown) reaching successive levels of the IM down to the papilla. AVRs arise at any level of the medulla. In the IM only few of them accompany the DVRs in tiny bundles. Most AVRs ascend as individual vessels distributed in a homogenous pattern outside the VB. Close to the border to the IS, they converge toward the entry into the VB of the IS traversing the IS within them, finally after partially joining in the OS they empty into the arcuate vein (blue) at the cortico-medullary border. AVRs draining the lower parts of the IS join the VB and ascend within the VB. AVRs from the middle and upper parts of the IS ascend as wavy vessels directly within the IBR of the IS towards the OS. They travers the OS as an ascending plexus that represents the main blood supply to the tubules (capillaries emerging from the EA are sparse), finally emptying into the arcuate vein. Some of them (only one is shown) ascend further into the medullary ray draining finally into an interlobular vein within the cortex. Note, the AVRs ascending within the VB establish togethers with the DVRs a VB-linked CCE system (enclosed by hatched lines). The AVRs ascending independently from the VB within the OM and IM are not part of this CCE system

 Short loops of Henle (Fig. 1a) belong to superficial and mid-cortical nephrons. They consist (i) of a thick descending part (proximal straight tubule PST) located within the medullary rays of the cortex and within the outer stripe (OS), (ii) of a thin descending limb (SDTL) that traverse the inner stripe (IS) within a VB, and after a turn into the (iii) thick ascending limb (TAL), which ascends through the inter-bundle region (IBR) of the IS, through the OS and the medullary rays [45, 56, 63, 67]. Touching its parent glomerulus with the macula densa, before transforming into the distal convoluted tubule (DCT).

 Long loops of Henle (Fig. 1a) belong to the juxtamedullary nephrons. Their loops consist of (i) a thick descending part (proximal straight tubule PST), which takes a tortuous descent through the OS, (ii) a thin descending limb (LDTL) which traverses the IS within the inter-bundle region (IBR), enters the IM and, at successive levels (details see below), turns (iii) into a thin ascending limb (ATL) which ascends through the IM, changing into (iv) the thick ascending limb (TAL) as it enters the IS, ascending further through the IS and OS reaching their parent glomerulus near the cortico-medullary border [45, 56, 67].

To facilitate description, we will consider three groups of long loops: the major group of “short” long loops (group A), turning back at variable distances shortly after entering the inner medulla, a smaller group of long loops (group B) reaching different levels of the middle part of the IM and a small population of “long” long loops (group C) that reach the inner third of the IM and the papilla.

 Collecting ducts (CDs) are established within the superficial cortex by the confluence of the nephrons [56]. A distal convoluted tubule (DCT) and a connecting tubule (CNT) mediate the transition from the loops. The CNTs of juxtamedullary nephrons join to arcades that ascend within in the cortex draining into CDs at superficial levels. Each CD accepts six nephrons. The cortical collecting ducts (CCDs) descend within the medullary rays and the OM (OMCDs) as unbranched tubes. Upon entering the IM (IMCDs) they start to join each other, after eight fusions they drain at the tip of the papilla into the renal pelvis.

## The vasculature of the renal medulla

The renal medulla (Fig. 1) is exclusively supplied by efferent arterioles (EA) of juxtamedullary glomeruli. They descend through the OS dividing into the descending vasa recta (DVRs) [34, 74, 102].

The DVRs together with the ascending vasa recta (AVRs) establish the vascular bundles (VBs) emerging in the OS, fully developed in the IS and decreasing in the IM. Only small side branches from DVRs contribute to the supply of the tubules of the OS. Most DVRs leave the VBs within IS to supply the dense capillary plexus of the middle and lower parts of the IS. A minor fraction of DVRs enters the IM, only few reach the papilla. They branch into sparse capillaries before passing over into AVRs.

AVRs draining the IM arise at any level, the longest in the papilla. Only a minority join the small VBs of the IM. The majority ascends in regular distribution among thin loop limbs and CDs towards the upper IM, where they converge to join the VBs of the IS traversing the IS within them.

**Fig. 2:**
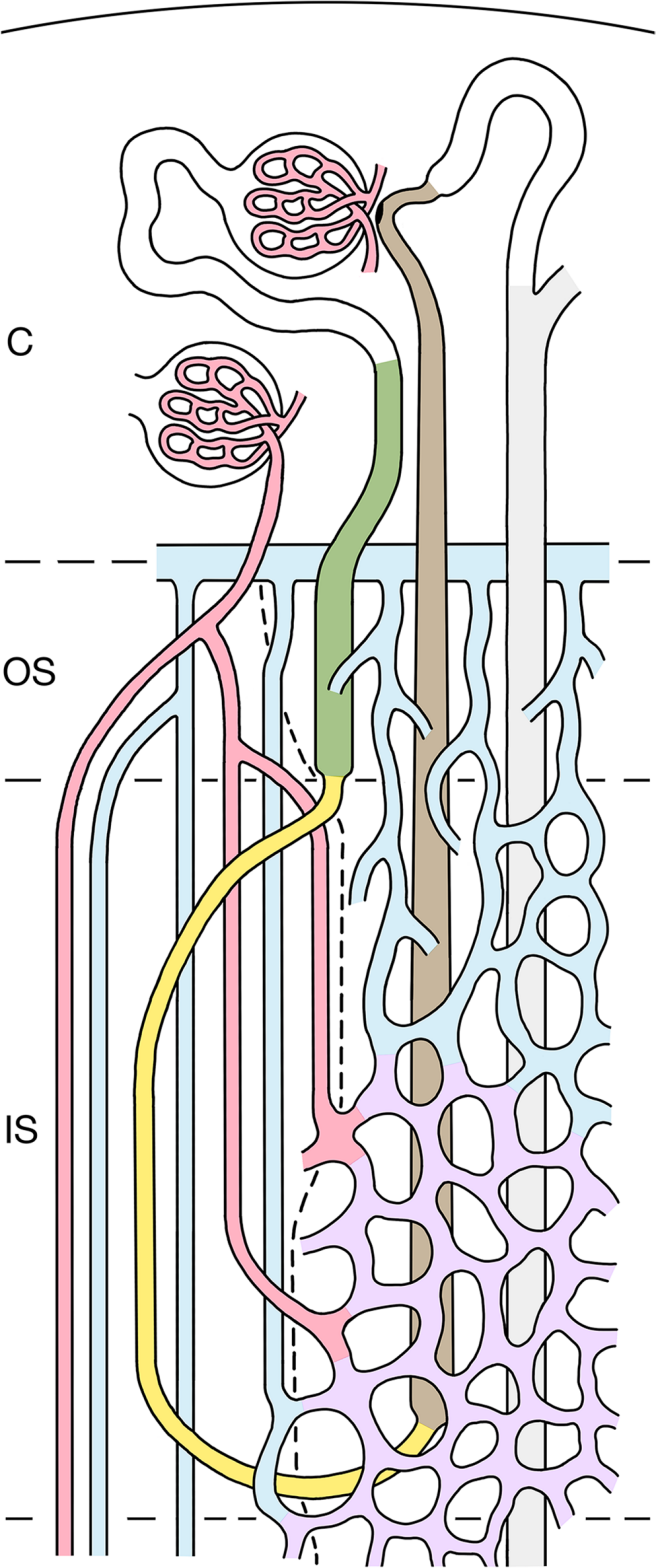
A simplified scheme of the outer medulla (OM). Only a short loop of Henle is depicted consisting of the PST (olive green) traversing the OS, the SDTL (yellow) descending through the IS integrated into the VB, turning close to the border to the IM into the TAL (brown), which ascends within the inter-bundle region (IBR) through the IS and OS, further through a medullary ray, passing the macula densa, finally draining via the distal convoluted and connecting tubule (DCT and CNT, white) into the collecting duct CD (grey), which descends parallel to the TAL, all together supplied by the respective capillary/AVR plexus of the IS and OS. The border between VB and the IBR is shown by a hatched line. The EA of a juxtamedullary glomerulus splits into DVRs (red) that descend within VB. Two of them leave the VB within the IS to supply the capillary plexus (purple) of the middle and deep portions of the IBR. Others descend further into the IM. AVRs (blue) drain the medulla. Those from the IM traverse the IS within the VBs in countercurrent position to DVRs (red) and SDTL(yellow). Those draining the capillary plexus of the lower portion of the IS join the VBs arranged in countercurrent position with DVRs that supply the IS. AVRs from the middle and upper portion of the IS ascend directly within the IBR of the IS and the OS, finally emptying at the cortico-medullary border into an arcuate vein (blue). Note that these AVRs form a dense ascending plexus traversing in countercurrent arrangement with PST and CD the upper parts of the IS and the OS

AVRs draining the capillary plexus of the IS display two patterns. Those emerging from the lower part of the IS join the VBs, those from the middle and upper part ascend directly within the IBR into the OS (Fig. 2).

Within the OS, the VBs fall apart. AVRs peeling off from the VBs and those ascending directly from the IBR of the IS traverse the OS as tortuous channels representing the dominant supply of the tubules. Finally, at the corticomedullary border, most AVRs empty into arcuate veins. Some ascend further within the medullary rays finally joining interlobular veins.

Note: AVRs from the IM and the lower part of the IS establish together with the DVRs a VB-linked CCE system, composed of the small VBs of the IM, the voluminous VBs of the IS and the remnant VBs of the OS. The AVRs directly ascending from the IS traversing the OS to empty directly into arcuate veins are excluded from this CCE system (Figs. 1b and 2).

## The regions of the medulla

### Outer stripe

**Fig. 3:**
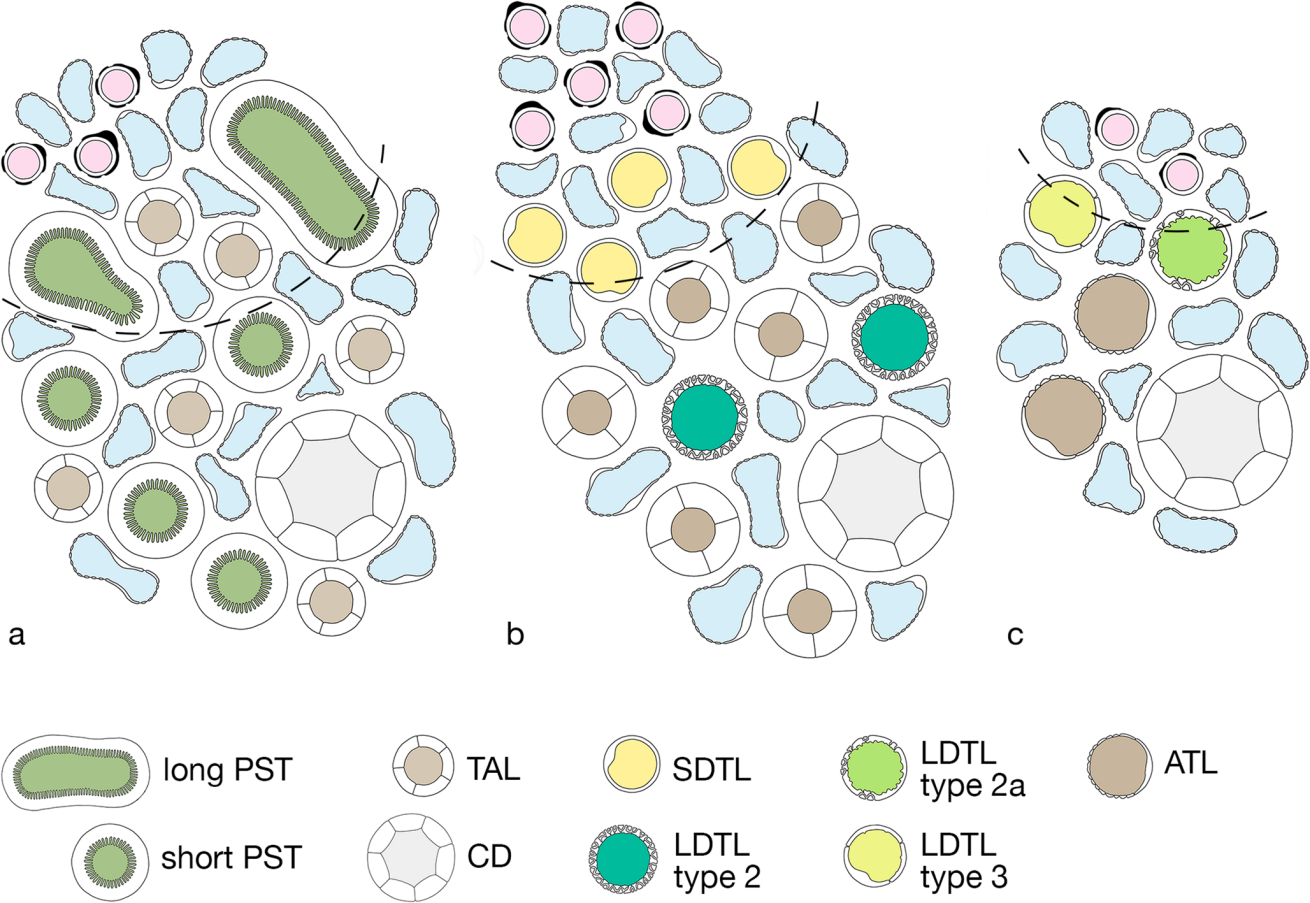
The regions of the rat medulla in schematic cross sections. The border between the vascular bundle (VB) including its adjacent tubules and the interbundle region (IBR) is delineated by a hatched line. The box may help to identify the various structures. **a **Outer stripe: A VB and the IBR are shown. The developing VB with DVRs (red) and AVRs (blue) is directly bordered by two PSTs (olive green) of long loops (shown in oblique profiles indicating their tortuous course) and the corresponding TALs (brown) are seen. More distant in the IBR 4 PSTs, 4 TALs of short loops and a CD (pale grey) are densely supplied mostly by AVRs. **b** Inner stripe: The VB has enlarged containing in its periphery 4 SDTLs (outlined by type 1 epithelium, yellow) associated with AVRs. In the IBR 2 LDTLs (outlined by type 2 epithelium, dark green) and in total 6 TALs and a CD are depicted embedded into a dense vascular plexus consisting of capillaries and AVRs. **c **Inner medulla (upper part): Outside the remnant VB 2 LDTLs (one type 2a in light green and one type 3 in yellowish green), 2 ATLs(brown) and a CD are homogenously associated with AVRs and capillaries

The OS arises in continuation of the medullary rays of the cortex [56, 63]. It contains the PSTs and TALs of the loops of Henle and the CDs. The PSTs of long loops, in contrast to their name “straight”, take a rather tortuous course making them the dominant tubular segment within the OS. The vasculature starts with EAs from juxtamedullary glomeruli splitting off into DVRs gathering together with AVRs to the small VBs of the OS. Like in the IS, VBs can be separated from an IBR (Figs. 2 and 3a). The PSTs of long loops are arranged close to the VBs, all other tubules are found in the IBR. Apart from sparse capillaries arising from DVRs, AVRs dominate the vasculature of the OS. Those coming up directly from the IBR of the IS establish together with those peeling off the VBs a dense ascending plexus in counter-current arrangement with PSTs and CDs. Interstitial spaces are extremely sparse, thus the contact between tubules and vessel is narrow. Of note: lymphatics are absent from the entire medulla [61].

### Inner stripe

The IS is subdivided into two regions [56], VBs and the IBR (Figs. 2 and 3b). The VBs are composed of DVRs, AVRs and the SDTLs [67]. The latter enter the VBs at the transition from the OS to the IS and leave them close to the IM, where they transform into TALs. The IBR contains the descending thin limbs of long loops LDTLs, the TALs of both loop types and the CDs. Together these tubules are embedded into a dense capillary plexus, which is supplied by DVRs that leave the VBs at middle and deep levels of the IS. The drainage of this plexus occurs by AVRs (Fig. 2). Those from the deep levels of the IS join the VBs, those from the middle and upper levels, gradually emerging from the capillary plexus, ascend as individual wavy vessels directly within the IBR into the OS forming a dense ascending plexus. Within the VBs, interstitial spaces are sparse, whereas in the IBR they are clearly wider, establishing together with the capillaries an AVRs the vascular-interstitial compartment (VIC).

### Inner medulla

The IM contains the descending and ascending thin limbs of the long loops (LDTLs, ATLs), representing one third of the total number of loops, the IMCDS including the terminal CDs and a specific microvascular system composed of DVRs, capillaries and AVRs [56, 67].

**Fig. 4:**
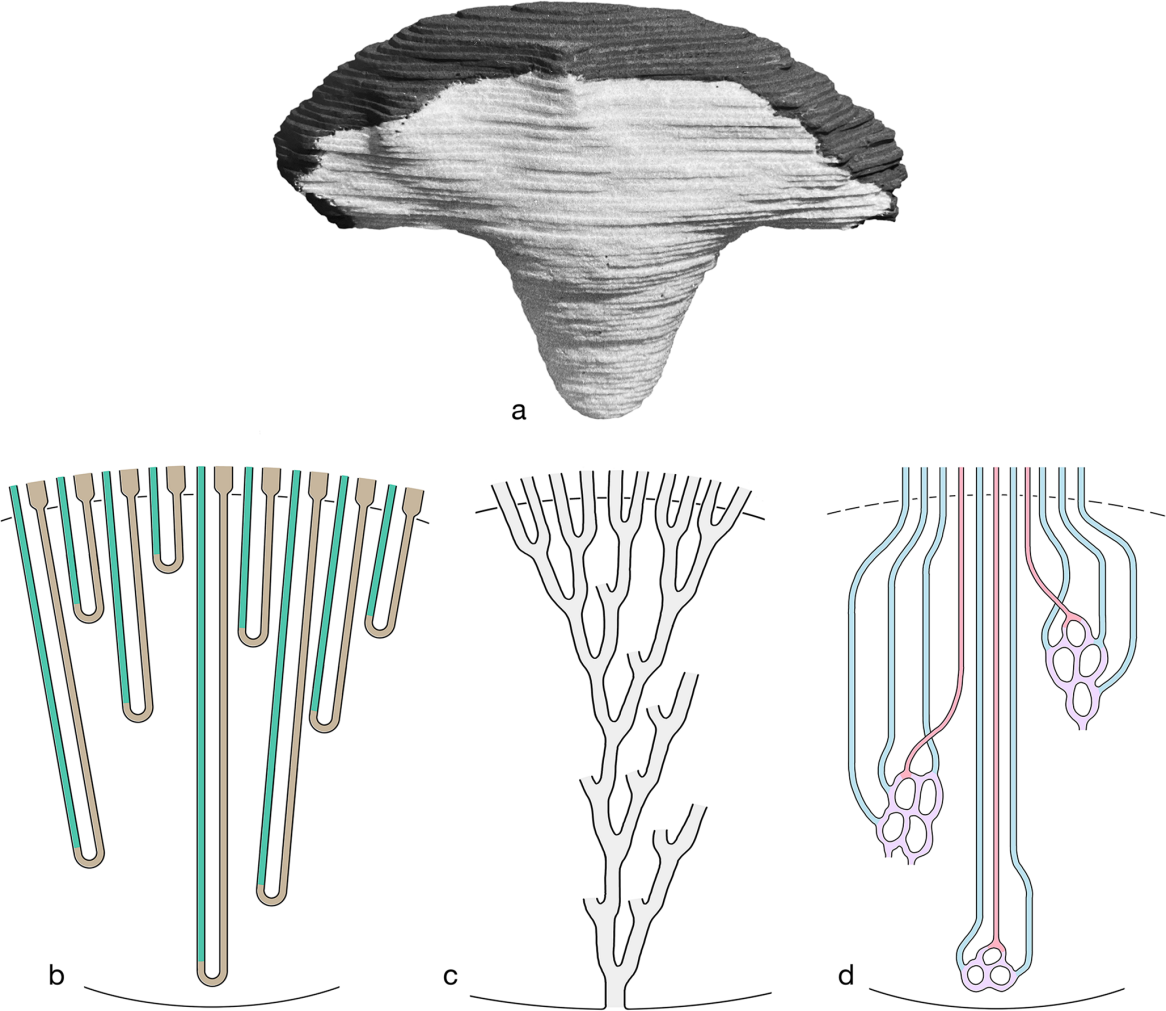
Composition of the IM.** a** Three-dimensional view of a reconstruction of the rat IM showing its shape as a pyramid. The light stained portion faces the pelvic cavity, the dark stained portion marks the entrance area from the IS. According to this shape all structures within the IM decrease toward the tip. From Becker 1978 [12]. **b** Schematic of the distribution of the long loops in the IM that form their bends at successive levels strongly decreasing in number toward the tip. DTLs are shown in green, ATLs in brown. Note that all loops have a prebend segment outlined by the ATL epithelium (brown), forming together with the corresponding portion of the ATL and the connecting piece the bend segment. **c** Schematic of the behavior of CDs that after entry into the IM join eight times before emptying into the pelvis. **d** Schematic of the vasa recta of one VB. DVRs (red) rapidly decrease in number only single ones reach the papilla. AVRs (blue) greatly outnumber the DVRs. They originate at any level of the IM from a sparse capillary network (purple). Only few of them ascend in association with DVRs within the VB. The majority ascends in homogenous distribution among loop limbs and CDs toward the border to the IS, where they converge to join the respective VB

The inner medulla tapers from a broad basis to a tiny papilla [12, 41]. This shape (Fig. 4a) reflects the rapid decrease of the numbers of loops of Henle, vasa recta and collecting ducts from the base to the tip of the papilla. An estimated number of 10,000 long loops enter the IM in the rat [12, 41], turning at successive levels continuously decreasing in number downwards (Fig. 4b). Only about 1,500 enter the papillary region and only a few of these reach the papillary tip [12, 41]. Similar values have been found by Layton and colleagues [70]. The CDs (Fig. 4c) upon entry in the IM continuously fuse, in total 8 times. Thus, from roughly 5000 CDs entering the IM less than 20 open into the renal pelvis. The ratio of ATLs to CDs decreases in the IM from about 2,5 at the base to 1,0 in the papilla [50].

The vasculature is shown in Fig. 4d. A minor fraction of DVRs enters the IM, in the upper portion of the IM still gathered in small VBs. On descending in the IM, the VBs continuously decrease in size, reflecting the decreasing numbers of DVRs and AVRs. In the lower half of the IM, VBs are no longer discernible, however individual DVRs reach the tip of the papilla. At successive levels, the DVRs split into sparse capillaries giving finally rise to AVRs.

AVRs represent the dominant vessel type of the IM. Only few ascend within the small VBs. Most AVRs ascend as individual unbranched vessels homogenously arranged among loop limbs and CDs towards the border to the IS, where they converge to join the VBs traversing within them the IS [41, 95, 97, 102]. AVRs greatly outnumber DVRs, ratios from 3 to 6 are reported [39, 95, 127], considerably decreasing flow velocity compared to that in DVRs.

**Fig. 5:**
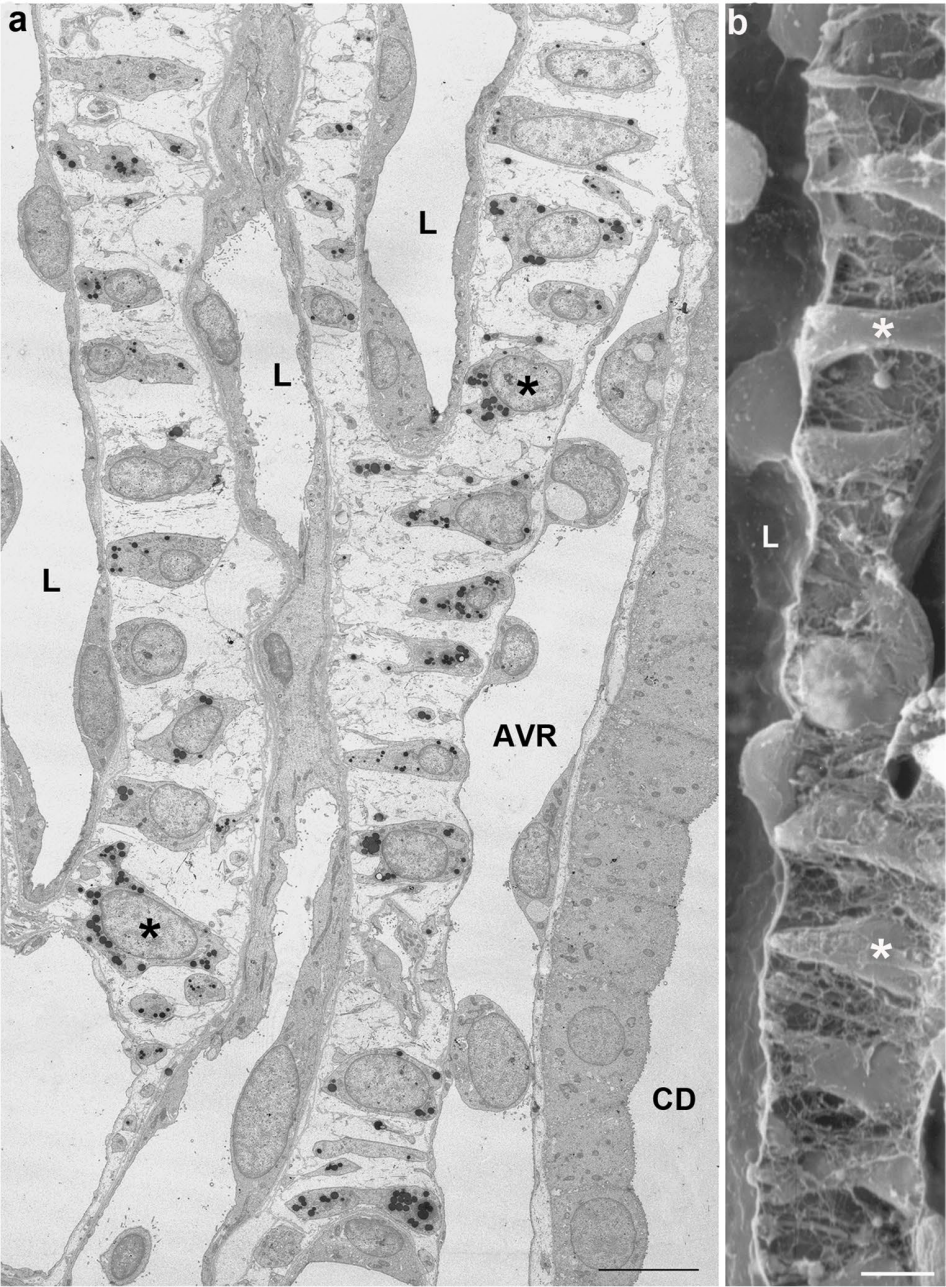
Lipid laden interstitial cells in the IM (rat). Longitudinal sections through the IM of the rat showing the ladder-like arrangement of the lipid laden interstitial cells (asterisks) separating the interstitial space into transversal micro-compartments, in **a** between loop limbs (L), an AVR and a CD, in **b** between a loop limb (L) at the left and an AVR at the right. **a** Transmission electron microscopy, Bar: 10 μm, **b** Scanning electron microscopy, Bar: 1 μm

In contrast to the OS and the IS, the IM displays wide interstitial spaces and a specific type of interstitial cells, the lipid laden interstitial cells [14] (Fig. 5). These cells are arranged like the rungs of a ladder spanning between loop limbs, AVRs and CDs. They create a horizontal compartmentalization of the interstitial spaces [75] largely separated from the compartments below and above, thus limiting longitudinal solute diffusion.

The prominent lipid granules are generally thought to be associated with the synthesis and secretion of some pressure active hormone most likely medullipin [32, 68, 82].

**Fig. 6:**
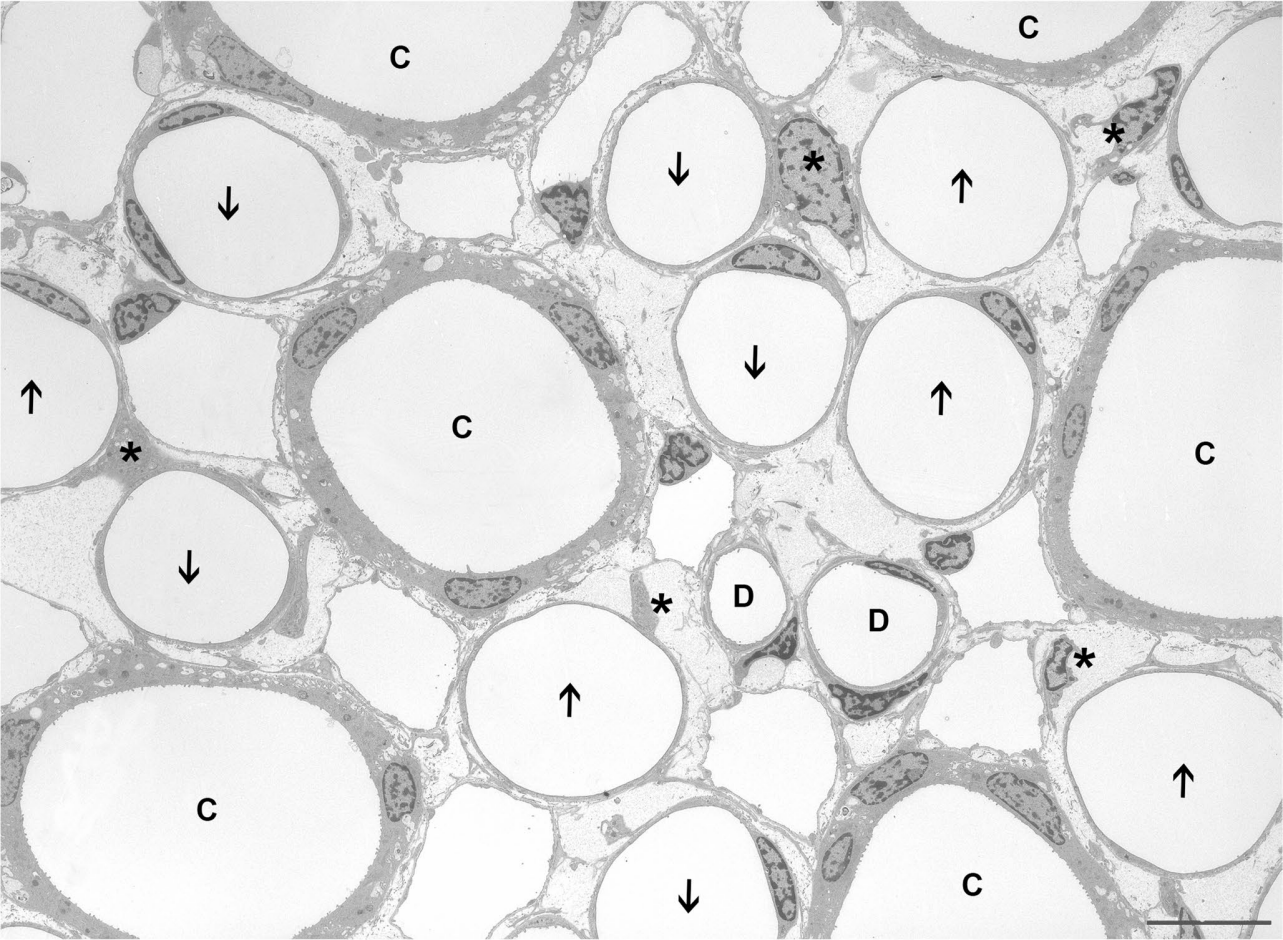
Cross section through the upper IM. Shown are profiles of 5 CDs(C), 5DTLs (arrows down), 5ATLs (arrows up), 2 DVRs(D), multiple AVRs/capillaries (easily discernible on the thin endothelium) and several interstitial cells (asterisks). Note the wide interstitial spaces. Regular structural relationships of tubules are only given to AVRs. Rat, TEM, Bar: 10 μm

A cross-sectional compartmentation is not as obvious in the IM as in the IS in light micrographs (Figs. 3c and 6). In meticulous work based on immunocytochemical overviews an intra-cluster and an inter-cluster region have been distinguished (summarized in [23]) roughly seen as continuations of the IBR and the VB of the IS, respectively. Intra-cluster regions contain CDs, ATLs and networks of AVRs. The inter-cluster regions contain DVRs and AVRs, DTLs and ATLs. We hesitate to accept that these regions are sufficiently separated from each other to maintain different interstitial environmental conditions.

In our view, the homogenous distribution of ATLs and of AVRs throughout the IM [95–97] together with the wide interstitial spaces suggests that the IM as a whole may be considered as a giant countercurrent system that allows CCE between all descending (DVRs, descending capillaries, DTLs, CDs) and all ascending structures (AVRs, ascending capillaries, ATLs) dependent only on the transport characteristics of the individual structures. The only exception may consist of the VBs in the upper most part of the IM in the sense that in agreement with [23] direct countercurrent exchanges between DVRs and AVRs in these remnant bundles may be favored compared to those among all other tubes.

## Cellular ultrastructure and transport characteristics of the involved tubular segments

### Loops of Henle

Thick descending limbs (PSTs) [21, 45], are equipped with a powerful transporting epithelium relevant in the present context because of its constitutive water reabsorption through leaky junctions and abundant water channels (AQP1) in both membranes. This increases the luminal solute concentrations, including salt and urea. The relevance of additional uptake of urea into PSTs by facilitated diffusion [7, 124] is unclear.

**Fig. 7:**
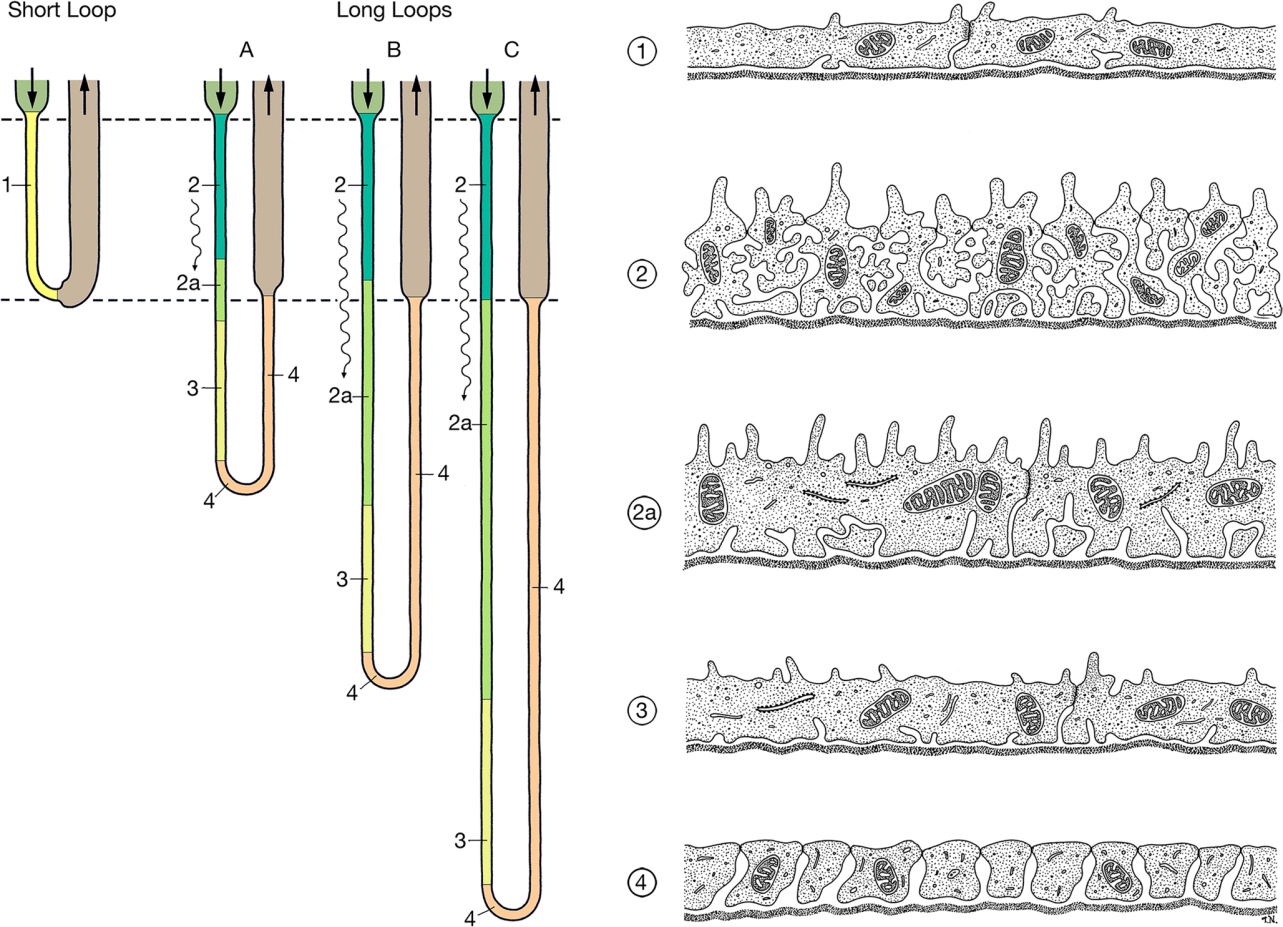
Thin limbs of Henle’s loop. Schematics of the epithelial structure and distribution. Five different epithelia are found among thin limbs in rat. Type 1, a flat and simply organized epithelium, with junctions of intermediate depth, only found in SDTLs (shown in yellow). Type 2, highly complex heavily interdigitating epithelium with dense apical microvilli, leaky junctions and basolateral infoldings forming a labyrinth of extracellular spaces with the cell body, many mitochondria, found in the initial portions of LDTLs in the IS (shown in dark green). Type 2a represents a less complexly organized version of type 2 epithelium, found in some loop limbs already in the IS and in loop limbs in the upper and middle portions of the IM (shown in light green). The transition from type 2 to type 2a epithelium is gradual. Type 3 epithelium, a simple epithelium with junctions of intermediate depth and basal infoldings, found in the terminal portions of all LDTLs (shown in yellowish green). Type 4 epithelium is a heavily interdigitating epithelium with leaky junctions. It is already found within the pre-bend segments of the LDTLs and is characteristic for the ATL (shown in brown)

 Thin descending limbs of short loops (SDTLs) [21, 45, 110] (Fig. 7) are made up of a thin simply structured epithelium (type 1 epithelium) with junctions of intermediate density and a sparse equipment with organelles. According to studies in the mouse the initial part of the of SDTLs is sparsely equipped with AQP1 channels [125]. The lower parts of SDTLs contain the urea transporter UT-A2 [7, 87, 120].

 Thin descending limbs of long loops (LDTLs) (Fig. 7). Originally, two segments were separated, an upper segment outlined by type 2 epithelium and a lower segment outlined by type 3 epithelium [45, 63, 110]. Here we subdivide the upper segment into an upper portion outlined by type 2 epithelium and a lower portion outlined by epithelium type 2a. The LDTL is water permeable down to the transition to the Type 3 epithelium [99].

**Fig. 8:**
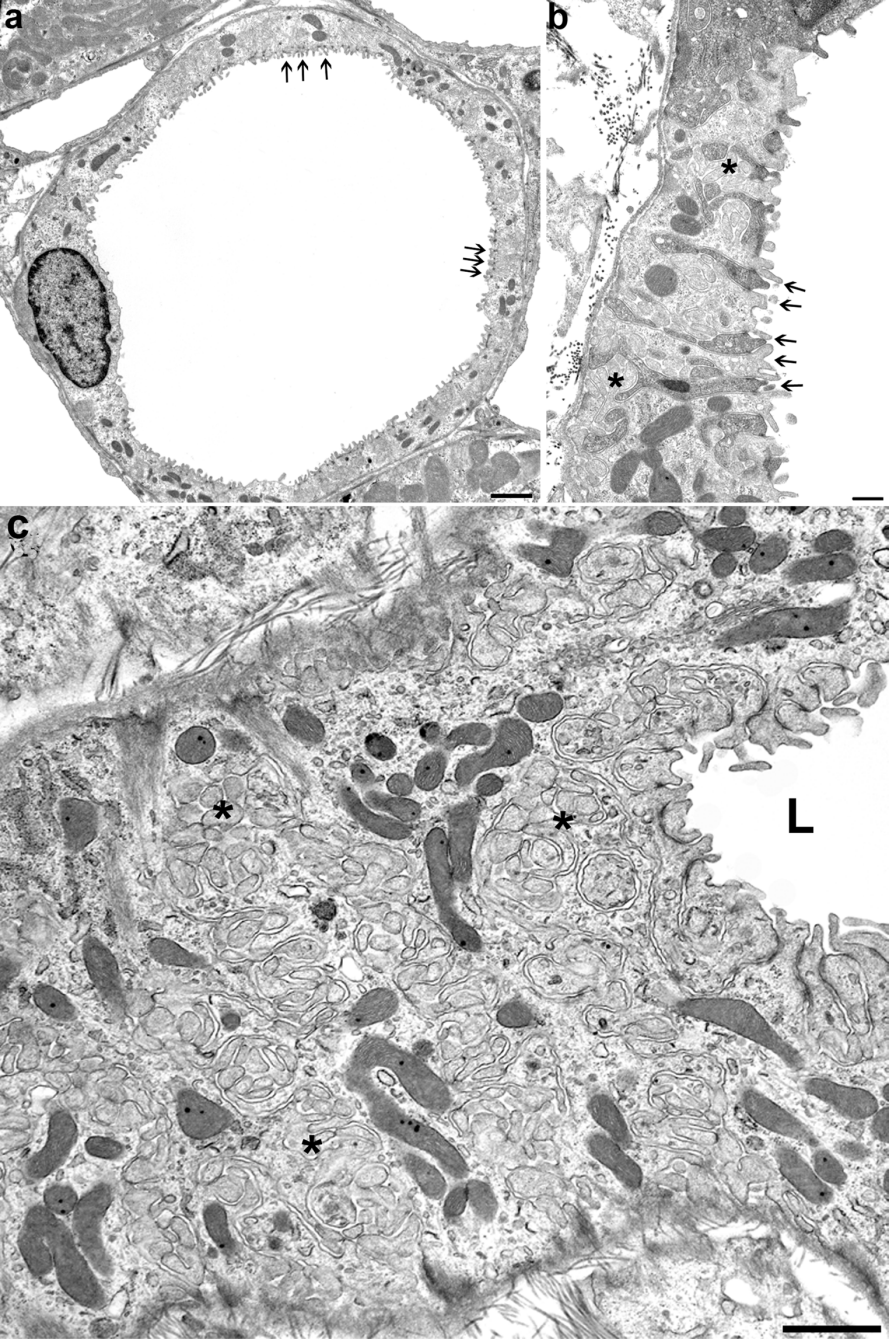
LDTL, epithelium type 2.** a** Overview showing the complex, comparably high epithelium with extensive interdigitations (arrows) and microvilli throughout the entire circumference. TEM, bar:10 μm. **b** Epithelial detail showing more clearly the many microvilli, the low junctions (arrows) and the extensive amplification of the basolateral cell membrane (asterisks) distributed within the entire epithelial body. TEM, bar: 2 μm. **c** Gracing section through the epithelium showing on the right the lumen (L) and the epithelial surface, on the left the major part the cytoplasm with the inclusion of huge amounts of convoluted assemblies of lateral cell projections (asterisks) amplifying the basolateral cell membrane. Note the many mitochondria. TEM, bar: 5 μm

 Type 2 epithelium [45, 63, 110] (Figs. 7 and 8) has a unique cellular organisation. First, it is characterized by an extremely high degree of cellular interdigitation, originally described by Zimmermann in 1911 [128]. The tight junctions consist of one junctional strand thus the junctions are leaky and the number of junctional channels is extensively increased by the tortuosity of the junction. Second, the lateral cellular spaces are extensively enlarged and filled with an elaborate basolateral labyrinth, presenting as convoluted assemblies of lateral cell projections.

The luminal surface is strongly amplified by densely arranged microvilli and both luminal and basolateral membranes exhibit a strikingly high density of uniform intramembranous particles. Numerous mitochondria are found in apical and basal positions. Such a kind of epithelial structure is not found in any other nephron segment. It resembles the salt secreting epithelium in the avian salt gland [30].

Cytochemical and immunohistochemical studies have revealed that the type 2 epithelium exhibits a sodium-potassium ATPase in both, the luminal and basolateral membranes [31, 46, 78] suggesting active salt secretion. Transports by this epithelium are sensitive to ADH among several other peptide hormones [24]. Moreover, salt may be transported through the tight junctions, which contain claudin 2 [29, 47]. The epithelium is highly permeable to water [19, 49] due to the abundance of the constitutive water channel aquaporin 1 (AQP1) in both membranes [86, 125].Unfortunately, the transport properties of this specific loop segment have never been studied by micro-puncture, thus our conclusions are necessarily solely based on the cellular endowment.

The elaboration of type 2 epithelium varies with the final length of the loops: the longer the loop the more complex seems to be its elaboration in the IS.

#### Type 2a epithelium

The type 2 epithelium decreases progressively in complexity along the descent of the loops, and we term the simplified type epithelium type 2a (Fig. 7) that precedes the transition into type 3. Compared to type 2, the type 2a epithelium shows decreased outer tubular diameter, epithelial thickness, number of mitochondria, apical microvilli and basolateral interdigitation. The density of intramembrane particles is reduced but still high [45, 110]. The structural simplification suggests a low capacity for salt secretion in type 2a. Because the dense particle density in both membranes, likely including the water channels, the segment continues to be highly permeable for water. This agrees with previous findings by micropuncture [99]. The change from type 2 to type 2a is gradual, from type 2a to type 3 abrupt when judged on the sharp decrease of the particle density in both membranes in type 3 [109].

Type 3 epithelium of the lower part of LDTL (Fig. 7) is simply structured consisting of relatively flat, non-interdigitating cells bearing sparse microvilli [45] and covered by an unusually thick surface coat [110]. The tight junctions are significantly deeper than in the upper segments. The spaces between the basal infoldings are not continuous with the lateral intercellular spaces, thus they are not part of a paracellular pathway route. The membranes lack the dense intramembrane particle density [45, 109] of the former segments suggesting low water permeability. This agrees with the very low density of aquaporin 1 (AQD1) channels in the terminal segments of LDTLs of all loops [22, 70, 89, 93].

The change from the type 3 epithelium to the type 4 epithelium (Fig. 7) [45, 110] is abrupt and starts before the bend (165 μm in average [23]). The prebend segment, the corresponding segment of the ATL and the segment connecting both may be summarized as “bend segments”, where net salt efflux occurs [98].

Thin ascending thin limbs (ATLs) are equipped with the type 4 epithelium (Fig. 7) [45, 63, 110] characterized by very flat but heavily interdigitating cells joined by shallow tight junctions, consisting of only one but prominent junctional strand. It is endowed with the chloride channel ClC-K1 [70, 93, 117]; aquaporins are completely lacking. Thus, the ascending thin limb is impermeable for water and likely little permeable for urea, but highly permeable for Cl^−^ and Na^+^ [22] possibly related to the expression of claudin 4 [47]. The high ion permeability of the epithelium has also been shown by micro-puncture studies [38, 40].

Conflicting data have been published concerning urea permeabilities [70, 72, 93]. Since no urea transporter has been found in any thin limb segment in the IM [83] urea transports in thin limbs do not seem to play an essential role in the concentrating process in the IM.

Thick ascending limbs (TAL) are equipped with a transporting epithelium endowed with a dense accumulation of Na-K-ATPases in the amplified basolateral membrane accounting for the powerful re-absorptive capacity of NaCl mediated by the Na^+^,K^+^,2 Cl^−^ (NKCC2) symporter in the luminal membrane [17], stimulated by ADH [37, 81, 105]. The density of Na-K-ATPase in the TAL exceeds by far that in proximal tubules [28]. The epithelium contains no water channels and also the tight junctions consisting of several strands seem to be fairly impermeable for water.

The thickness of the TAL-epithelium (likely an equivalent of the transport capacity) decreases in flow direction [50]; the thickest portions are found at the very beginning in the IS [4, 6, 60]. Short loops have at all levels a thicker TAL-epithelium than long loops [3]. The elaboration of the TAL-epithelium is ADH dependent [4, 6, 60, 115].

The cortical TALs are functionally different from the medullary TALs in a variety of aspects [3, 37]. Important in the present context: the transport capacity for Na + is decreased, [18].

Collecting ducts (CDs) are equipped with a heterogenous epithelium consisting of two cell types, principal cells and intercalated cells. In the present context, only the principal cells are relevant [45, 63]. The thickness of principal cells increases along the descent from the cortex to the papillary tip, from flat in the cortex (CCDs) and outer medulla (OMCDs) to cuboidal in the IM (IMCDs) [21]. The cells are connected to each other or to intercalated cells by deep tight junctions consisting of several densely arranged anastomosing strands providing low permeability for ions and water. The reabsorption of sodium is driven by abundant Na-K-ATPases located in the lateral and basal membranes in conjunction with the aldosterone sensitive sodium channel, ENaC in the luminal membrane [84].

In the present context the machinery regulating the reabsorption of water through principal cells is of utmost importance [80]. The transporters are aquaporins differently distributed in both membranes [15, 48, 52, 103, 119]. The basolateral membranes contain the constitutive aquaporins AQP3 and AQP4. AQP2 channels are stored in the apical cytoplasm in longitudinal vesicles called aggrephores. In the presence of ADH these vesicles fuse with the luminal membrane, converting the luminal membrane from water-impermeable to water-permeable [85].

The principal cells of the innermost third of the IMCD play a decisive role in the urine concentrating process. They contain the ADH-sensitive urea transporters UT-A1/3 in the apical membrane and possibly UT-A4 in the basolateral membrane [8] allowing facilitated urea reabsorption into the papillary part of the IM. The abundance of UT-A 1/3 in the apical membrane is rate-limiting for this process [88, 124].

## Microanatomy of medullary vessels

Juxtamedullary EAs are larger in diameter than cortical efferent arterioles; their size even exceeds that of their corresponding afferent arterioles. They display two to four layers of smooth muscle cells, their contractile power seems to be significant [26, 59, 62].

In the DVRs the smooth-muscle cells are gradually replaced by pericytes, which form an incomplete layer around the endothelium [25]. Their dense assemblies of microfilaments imply a contractile function. In contrast to smooth muscle cells, they are not contacted by nerve terminals. The thin continuous endothelium expresses in its luminal and basal membranes the water channel AQP1 [89] and the urea transporter UT-B1 [116] and is permeable to NaCl [92]. Finally, the DVRs lose their pericytes, and the concurrent appearance of endothelial fenestrations marks their gradual transformation into medullary capillaries.

The ultrastructure of the peritubular capillaries in the kidney are of the fenestrated type, identical in the cortex and the medulla. They consist of an extremely flat endothelium surrounded by a thin basement membrane. In non-nuclear regions, the endothelial cells contain densely and regularly arranged fenestrations that are bridged by a thin diaphragm. An estimated 50% of the capillary circumference is composed of these fenestrated areas [26]. The diaphragm consists of a very thin (5–6 nm) single-layered proteinaceous membrane [11] that accounts for the high permeability to water and hydrophilic solutes.

The wall structure of the AVRs is identical to that of capillaries [26, 57, 74].They are bounded for their entire length by an extremely flat endothelium with extensive fenestrations lying on a thin basement membrane.

AVRs and capillaries because of their diaphragm-bridged fenestrations are highly permeable to water, ions, and small water-soluble solutes including urea [77, 92]. Therefore, we may expect full equilibration between these vessels and the interstitial space and consider both together as the vascular-interstitial compartment (VIC).

## The urine concentrating function: a preface

The urine concentrating mechanism can be subdivided into a basic process in the OM by which urine is concentrated to a height of about 600mosmol/L and a final process in the IM, by which urine concentrations up to 1200 mosmol/L are reached in man and 3000 mosmol/L in rat [104]. Both mechanisms depend on each other.

We will first describe the process in the OM in a model that omits the mechanisms concerning the IM, second the mechanism in the IM and finally a summary of both.

## The basic urine concentrating mechanism in the rat outer medulla

The OM consists of the OS and IS. Within both VBs and the IBR are distinguished (Figs. 2 and 3a). The VBs function as a CCE between DVRs and AVRs, in the IS they include the SDTLs as a second descending tube.

**Fig. 9:**
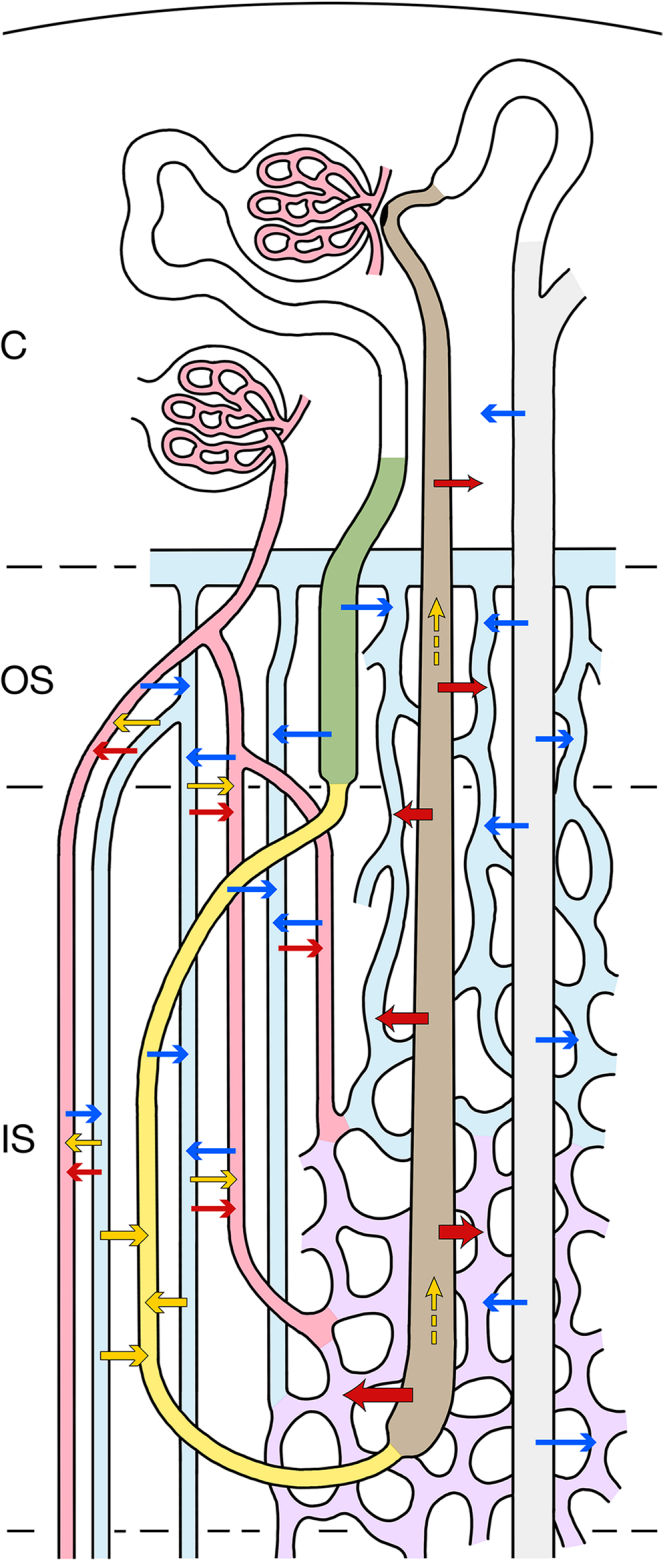
Schematic explaining the basic urine concentration in the OM. A mid-cortical nephron with a short loop of Henle composed of a PST (olive green), a SDTL (yellow), a TAL (brown) and a CD (grey) are shown together with the supplying vessels, DVRs (red), capillaries (purple), AVRs and the arcuate vein (blue). A long loop of Henle is omitted. **Function**: Red, yellow and blue arrows indicate transports of salt, urea and water, respectively. Thick black-rimmed arrows indicate active or facilitated transport, thin arrows transports through channels or simply diffusion, hatched arrows transports with flow. The basic mechanism of urine concentration consists of dumping salt by the TAL into the VIC of the OS and IS including AVRs and capillaries. This mechanism is most powerful in the deepest, the initial segment of the TAL [4, 6, 60]. Moderate amounts of salt and urea are added by DVRs. Urine concentration starts by osmotic water extraction from PSTs and CDs into AVRs within the OS and the upper part of the IS, continued within the lower part of the IS into the capillary plexus. Water extraction from the upper part of the SDTLs contributes to total water reabsorption within the OM. AVRs coming up from the IM traverse the IS with the VBs undergoing CCE (concerning salt, urea and water) with DVRs as well as with the SDTL leading to uptake of urea resulting in bypassing the VIC of the OM by outflow within the TAL. AVRs draining the capillary plexus of the lower part of the IS join the VBs becoming part of the VB-linked CCE system minimizing the loss of solutes by CCE with DVRs. The directly ascending AVRs from the upper part of the IS are excluded from this system

The IBR in the OS contains the PSTs, the TALs and the CDs, dominantly supplied by AVRs. The IBR of the IS contains the LDTLs, the TALs and the CDs embedded in a capillary/AVRs plexus that is supplied by DVRs leaving the bundles within the middle and lower parts of the IS. In the lower part of the IS the dense capillary plexus is drained by AVRs that join the VB. In the middle and upper parts of the IS capillaries become progressively integrated into a mixed plexus of capillaries and AVRs that directly ascends through the IS and continues throughout the OS (Fig. 9).

Due to the leaky endothelia, capillaries and AVRs are in complete equilibrium with the surrounding interstitial spaces, representing together the combined vascular-interstitial compartment (VIC) of IS and OS.

The re-absorptive function of TALs represents the decisive basis for the urinary concentrating process. Receiving a salt rich tubular fluid from SDTLs, they stimulated by ADH [37, 51] dump salt [17] into the VIC of the OM, leaving behind the water. The latter is carried up into the cortex and, in the presence of ADH, recovered there into the general circulation through osmotic withdrawal from the CNT [55] and CCD.

The TALs undergo alterations along their ascent in the OM. The thickness and the complexity of the epithelium are highest in the deepest part, and gradually decrease through the OM and the medullary rays [6]. Thus, the most powerful reabsorption achieving the highest interstitial salt concentrations occurs within the deepest region of the IS creating the basis for the salt gradient within the OM [4, 6, 60]. Urea coming up with AVRs from the IM contributes to a minor extend to the osmolality within the VIC of the OM, the major part bypasses the VIC by uptake into the SDTLs (see below together with the concentrating mechanism in the IM).

The permanent addition of salt to the VIC by TALs warrants that at each level of the OM the total osmotic concentration in the VIC is higher than in the descending limbs and CDs and permits at each level water extraction from both.

The urine concentrating process starts in the OS by water reabsorption from PSTs and CDs efficiently operating by CCE with AVRs. Since the PSTs of long loops take a tortuous course through the OS, the gain is likely high. Water reabsorption is continued in the IS, first to a minor degree, within the VB between AVRs and the initial portions of SDTLs, which are equipped with the water channel AQP1 [125]. Second and effectively between the CDs and the directly ascending AVRs, continued within the deeper parts of the IS with the capillary plexus, resulting in a twofold increase of urine concentration in CDs (some 600mosmol/L).

To reach this concentration 50% of the water must be reabsorbed within the OM, of which the major part is likely extracted already from the initial parts of the CDs and the PSTs based on CCE of both with AVRs. This essentially decreases the inflow of water into the medulla. This may explain the outstanding topographical relationships between AVRs on the one side and PSTs and CDs on the other at their entry into the medulla.

The maintenance of the osmotic gradient in the OM is ensured by CCE within the VBs between the DVRs and AVRs supplying the middle and lower parts of the IS. Subject to CCE are salt [92], urea facilitated by the urea transporter UT-B1 [7, 116] and water promoted in DVRs by the water channel AQP1 [89]. The washout of solutes by AVRs is permanently balanced by additions of salt from TALs and urea from terminal CDs (see later).

This concept of the urine concentrating function within the OM is totally different from a mechanism based on CCM and agrees in essential aspects with the view of Anita and Harald Layton [69, 104]. Neither the structural situation nor the functional processes are compatible with the CCM hypothesis, details were already compiled above.

## The urine concentrating mechanism in the rat inner medulla

**Fig. 10:**
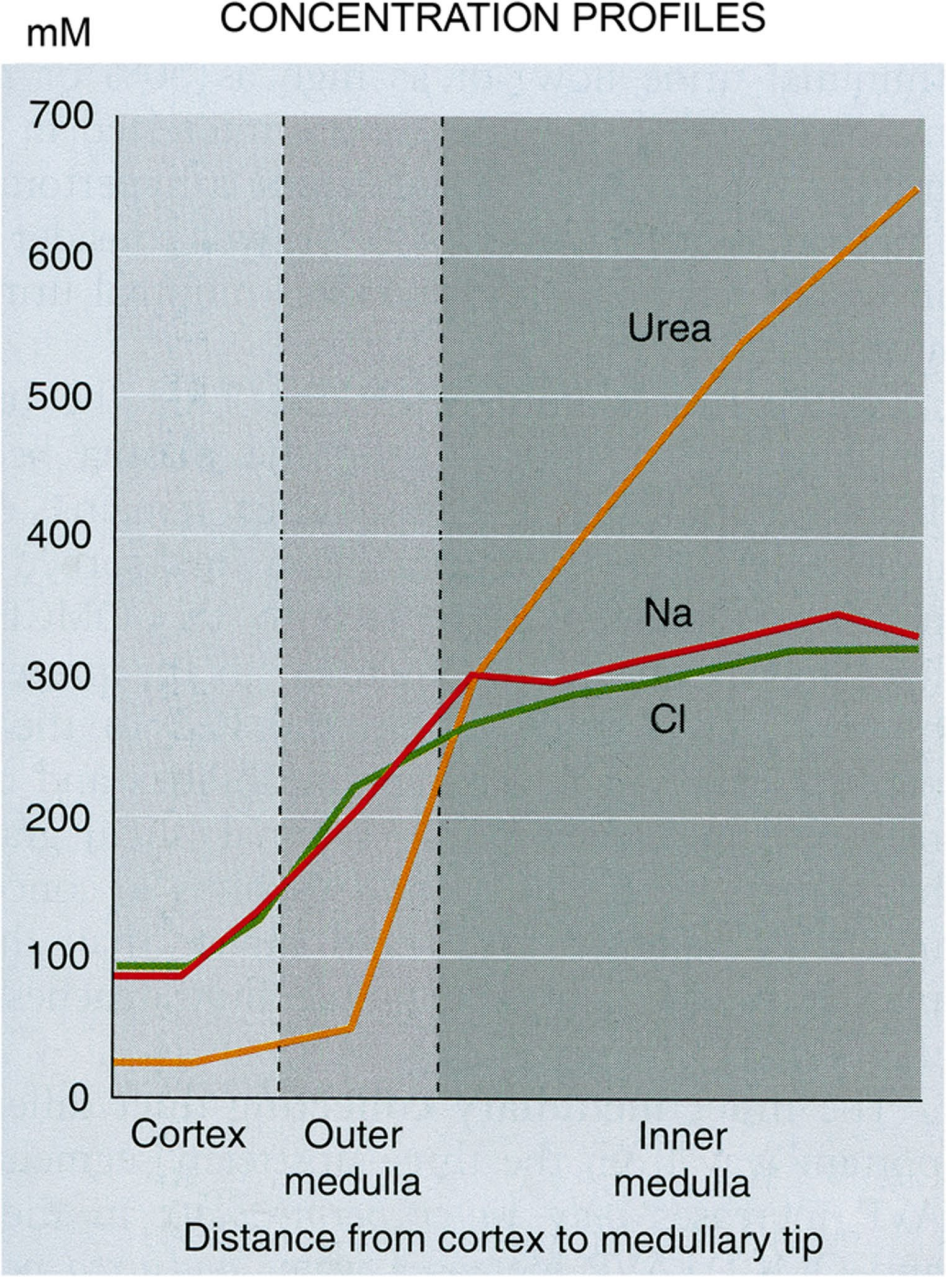
Increase of interstitial solute concentrations in the kidney during antidiuresis. Salt: From a base line in the cortex, the sodium concentration increases steeply within the OM, the further increase in the IM is flat. The chloride concentration parallels the sodium concentration. Urea: From a low concentration in the cortex the urea concentration rises steeply in the lower part of the OM (roughly corresponding to the IS). In contrast to salt it continues to increase steeply within the IM. From: Ullrich, Kramer, Boylan 1961 [118]

The mechanism is based on the accumulation of urea and salt and their distribution in a longitudinal gradient with the highest concentration of both in the papillary tip. The gradient is steep for urea, flat for salt [118] (Fig. 10).

The problem how the build-up of this dual gradient without up-hill transports is achieved has challenged generations of researchers since the seventies of the last century. Several “passive models” have been developed. The models published in 1972 by Kokko and Rector and by Stephenson [54, 112] are outstanding, they have greatly advanced our understanding of the problem and have highlighted the relevance of specific interactions but have never reached the level of a generally accepted mechanism. A detailed discussion is found in [23, 41].

More recently, immunohistochemical studies by Pannabecker, Layton and Dantzler [22, 23, 70, 71, 93–98, 104, 121], have extended our knowledge about the characteristics of inner medullary vascular and tubular components and their distribution within the IM and have initiated new approaches [70, 104]. These observations represent an essential basis for our proposal.

### The present concept

We propose that the salt and the urea gradients are generated without any active transports in the IM, instead by dragging of salt by LDTLs from the IS into the IM and by urea recycling via the terminal CDs, respectively. The resulting gradients are maintained by the VB-linked CCE system between AVRs and DVRs. We will first describe the establishment of the salt gradient, second the urea gradient.

### The salt gradient

The generation of the salt gradient in the IM is based on secretion of salt into LDTLs in the IS, followed by dragging of salt by the LDTLs into the IM, finally delivering the salt to successive levels of the IM according to the length of the loops. The bend segments represent the main sites of salt dumping into the interstitial space of the IM [42, 72, 98]. Uptake of salt into LDTLs in the IS, releasing the salt in the IM and bringing it back into the IS represents a kind of salt cycle, which had already previously been suggested based on micro-puncture-studies [24].

The LDTLs are loaded in the IS with a certain volume of a salt concentrated fluid likely adapted to the final concentration of the urine. We propose that this occurs by salt secretion and by the subsequent uptake of the corresponding amount of water. We suppose that the height of the load differs among short, intermediate and long LDTLs according to the elaboration of their type 2 epithelium. Thus, the longest LDTLs are loaded with the highest quantity of a salt enriched fluid. However, to reach the required high salt concentration in the papilla further concentrating steps are necessary along the descent down the IM.

**Fig. 11:**
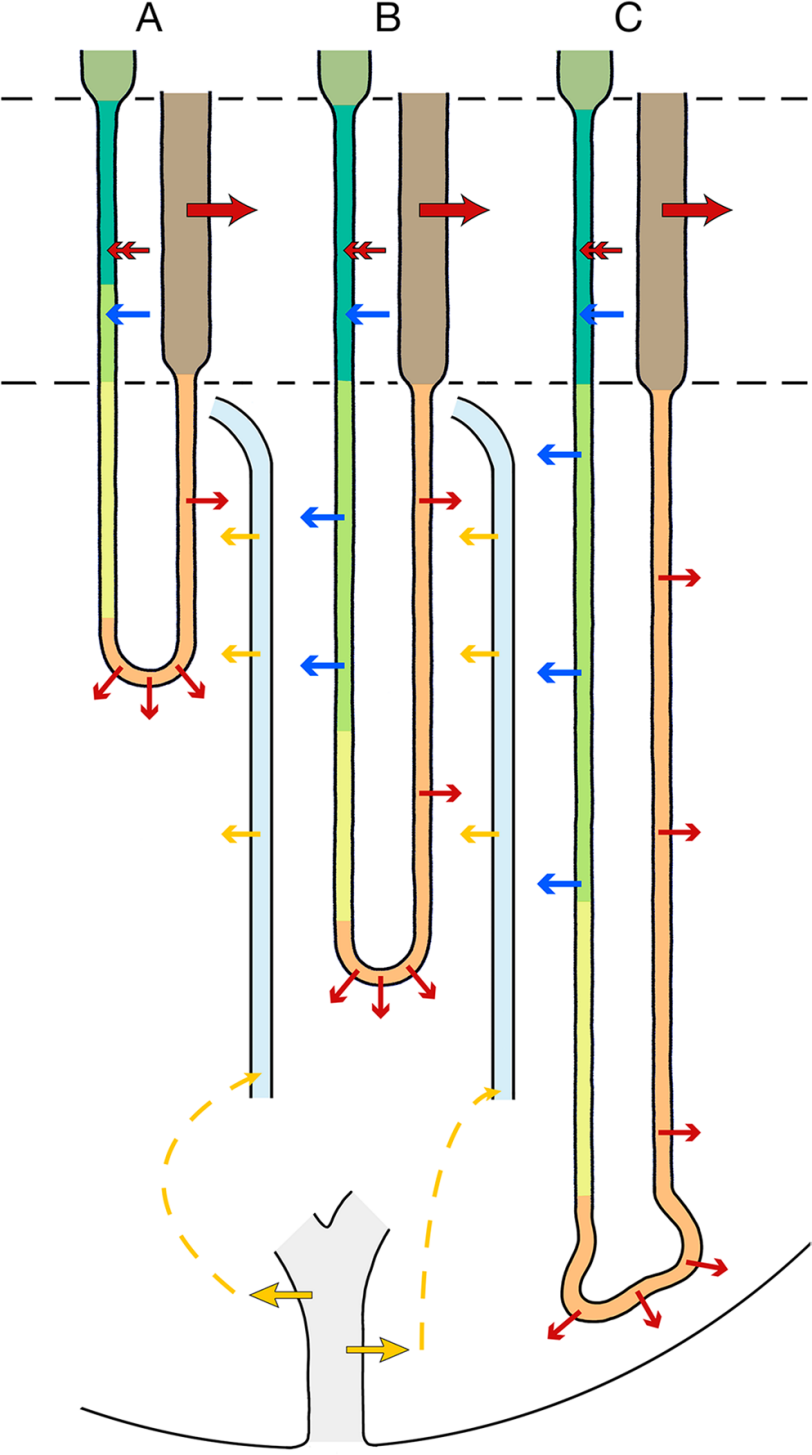
Salt cascade. Shown are: three LDTLs of group A, B and C, a terminal CD and AVRs starting in the papillary region turning at the border to the IS to empty in a VB (not shown). Type 2 epithelium is presented in dark green, type 2a in light green, type 3 in yellowish green, type 4 in light brown, ATLs in brown, TALs in dark brown, AVRs are shown in light blue. Black-rimmed red arrows indicate active salt reabsorption, double-headed red arrows salt secretion, thin red salt diffusion, blue arrows water uptake or extraction, black-rimmed yellow arrows facilitated urea release, thin yellow arrows urea diffusion. The basis for the cascade mechanism consists of salt reabsorption by TALs into the vascular-interstitial compartment (VIC) of the IS. LDTLs concentrate their luminal fluid in the IS by salt secretion (type 2 epithelium) and determine the quantity of the concentrated fluid by correlated water uptake. Group A LDTLs bring the salt enriched fluid through the type 3 segment down to the prebend segments within the upper part of the IM releasing the surplus of salt through the type 4 epithelium into the VIC, continued by ATLs. Together with urea from AVRs the interstitial osmolality at this level rises above the osmotic concentration within the group B LDTLs (type 2a epithelium) resulting in water extraction increasing their luminal salt concentration a second time. Through the subsequent type 3 segment, they bring down the salt enriched luminal fluid to the prebend segment within the middle part of the IM releasing the surplus of salt through the type 4 epithelium into the adjacent VIC. Again, together with urea from AVRs, the interstitial osmolality at this level rises, resulting in water extraction from the group C LDTLs (type 2a epithelium) increasing a second time their luminal salt concentration. Through the subsequent type 3 segment they bring down the salt enriched fluid to their bend segments releasing the surplus of salt by the type 4 epithelium into the papilla, achieving highest salt concentrations within the papillary VIC

We propose a cascade process (Fig. 11) among the LDTLs described for simplicity in three steps. In the first step the large group of short LDTLs (group A**)** dump salt into the interstitial space at the upper level of the IM, increasing together with urea released from AVRs the interstitial osmolality at this level. This enables in a second step water extraction from the smaller group of LDTLs of intermediate length (group B) increasing their luminal salt concentration. They will carry down the increased salt concentration to intermediate levels of the IM releasing the surplus of salt along the bend segments increasing again together with urea released from AVRs the interstitial osmolality at intermediate levels of the IM. This enables water extraction from the small **group C** of long LDTLs, which in the third step will bring the re-increased salt concentration down to the papillary region establishing there the highest salt concentrations.

This process resembles in essential aspects published in [20, 99].

### Precise description

LDTLs of all long loops are composed of several segments. They have a first segment in the IS that is basically equally structured in all LDTLs defined as type 2 epithelium [45, 110]. It simplifies in elaboration along its descent to type 2a. In LDTLs of group **A** loops the simplified type 2a epithelium is already found in the IS, the LDTLs of group **A** are already equipped with type 3 epithelium when they enter the IM. In group **B** loops the transition from type 2a to type 3 epithelium takes place at intermediates levels of the IM, in group **C** at even deeper levels. The type 3 segments eventually change into the prebend segments, which are outlined already by type 4 epithelium. This segmentation basically correlates with the description by Layton and colleagues [23, 70, 83].

Type 2 epithelium, due to its complex cellular elaboration and its water permeability accounts for the loading of LDTLs with a salt enriched fluid. The armament of the epithelium with an apical sodium pump coupled with water permeability accounts for active salt secretion increasing the luminal salt concentrations (details described above) followed by water uptake. The secretion of sodium into LDTLs is ADH sensitive [24], thus the entire process is regulated by ADH.

In the type 2a epithelium salt secretion is abolished or curtailed since the transitions are gradual, water permeability is maintained likely coupled with low sodium and urea permeability [70].

In the IM the major part of LDTLs is equipped with the type 3 epithelium, most of them likely belonging to type A loops. LDTLs equipped with type 2a epithelium in the IM (types B and C loops) decrease in number toward the papilla but few of them are still found in deep levels of the IM [110] suggesting that they belong to the longest long loops.

The short LDTLs of group A turning back in the upper region of the IM, likely outlined by type 3 epithelium, are impermeable to water (AQP1-negative) [22, 70, 97] and also to sodium and chloride (CIC-K1-negative) [117]. Thus, they likely act simply as conducting tubes bringing the salt enriched fluid provided within the IS down to the upper level of the IM. Releasing the surplus of salt from their bend segments will increase the salt concentration in the surrounding interstitial spaces to concentrations corresponding to luminal levels achieved by salt secretion in the IS.

On entering the IM, the LDTLs of group B and C loops that reach intermediate and deep levels of the IM are outlined on entering the IM by type 2a epithelium permeable to water but of low permeability to urea and salt [70]. This enables to increase their luminal salt concentration by osmotic water extraction due to higher osmolality in the surrounding interstitial space provided by salt release from the bend segments of the shorter long loops of group A and the addition of urea from upcoming AVRs (originating from reabsorption in the terminal CDs; see later).

The segments of **B** and **C** groups outlined by type 2a epithelium (that reach middle and deep levels of the IM) are followed by segments outlined by type 3 epithelium. Like the initial IM-segments of group **A** loops, they act as conducting segments carrying the salt enriched fluid down to their prebend segments in the deep IM including the papillary region. The fact that the longest long loops (group **C**) reaching the tip of the papilla have prominent bend segments elongated by long transversely running pieces [95–98] ensures strong salt release into the VIC of the papilla, the deepest point to start the CCE in the vascular system.

The increase in salt concentration in LDTLs along their descent in the IM likely parallels the increase of urea concentration in CDs. Less and less water has to be extracted from LDTLs and CDs to achieve the same increment in salt concentration in LDTLs and urea concentration in CDs. This reflective behavior of water extraction from CDs and LDTLs can be taken as evidence for the water extraction mechanism in LDTLs.

The ATLs have a uniform epithelium originally termed type 4, that is impermeable to water but highly permeable to sodium and chloride [38, 40, 117]. Since during their ascent the surrounding interstitial salt concentrations decreases, they will continuously release salt contributing to the actual interstitial salt concentrations at any IM level. Due to the larger inner diameter of ATLs compared to LDTLs [53] a lower flow velocity in ATLs may retard the decrease of the interstitial osmotic concentration toward the IS.

For simplicity, we have presented the salt dragging mechanism in a cascade of three groups of loops, short, intermediate and long long loops (Fig. 11). Loops turn back successively at any level, in a pattern that the number of bends, the hot spots of salt dumping gradually decrease towards the papilla correlated with the decrease in total volume of the IM [98].

Thus, the mechanism that we describe in three steps actually represents a process of a continuous series of steps, in which salt released from bends at any level increases the salt concentration in longer loops for delivery at deeper levels. The individual steps afford separation from each other likely provided by the transversely arranged interstitial cells confining longitudinal diffusions [75, 92, 98].

This system likely underlies the ability to adapt the salt transport down into the papilla to the necessity for the final urine concentration. The strength of salt secretion into LDTLs in the IS (under the control of ADH) will determine the volume and concentration of the salt enriched fluid that enters the IM and being processed in the stepwise increase in concentration to different depths in the IM. A low final urine concentration may need only few steps, a maximum final concentration all steps for increasing the salt concentration to the highest values in the longest long loops.

Increasing salt concentration in LDTLs down the IM by a cascade mechanism has first been proposed in 1983 by Kriz [58] and mathematically modelled in 1987 by Lory [76], based on assumed salt secretion into the upper parts of LDTLs in the IM. Instead, the present model considers water extraction from LDTLs in the IM, thus abstains from an active process. It agrees in essential features with a similar cascade model published in 1986 by Layton [71], as well as with the “pipe mode” hypothesis of salt dragging by Layton and colleagues in 2004 [70].

### The urea gradient

In contrast to the shallow salt gradient, urea in antidiuresis is accumulated in the IM in a steep longitudinal gradient for two purposes. As an end-product of the nitrogen metabolism urea is enriched in the urine and excreted. On the other hand, in the terminal CDs part of urea is diverted and due to the armament with the urea transporters UT-A1 and UT-A3 [88, 114, 124] released by facilitated diffusion into the VIC of the papilla creating there with the highest interstitial urea concentration the nidus for the urea gradient in the IM. Precondition for this diffusional process is that the luminal concentration of urea in the terminal IMCDs has reached higher levels than in the adjacent VIC. This is achieved by osmotic water extraction from the urea impermeable CDs along their descent down the IM. This needs an osmotic driving force that cannot be produced by urea alone. The necessary interstitial osmolality for water extraction from CDs along their entire length is reached only together with the amount of salt placed on top of urea (see below).

**Fig. 12:**
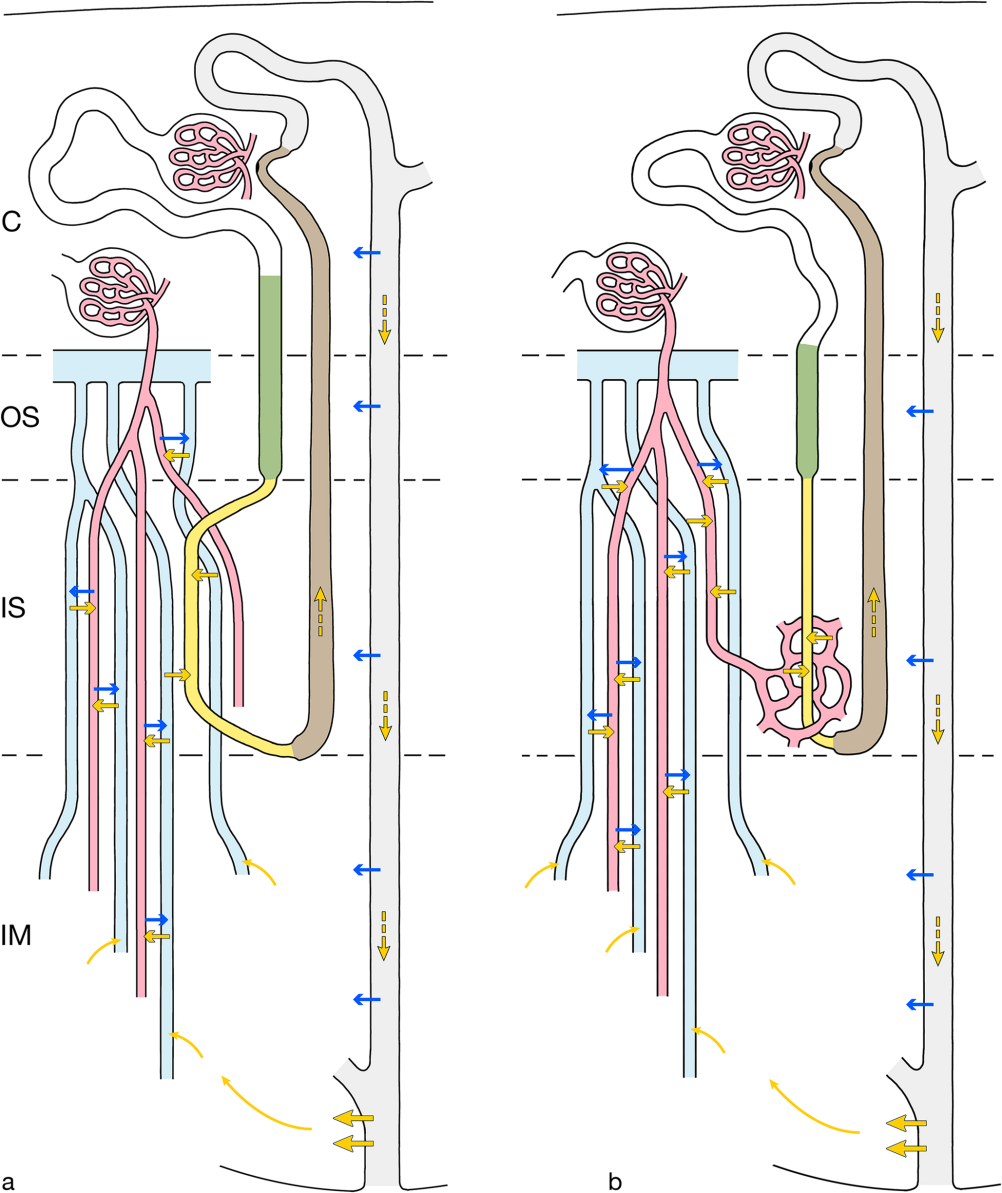
Urea recycling and trapping. A short-looped nephron, a CD, a VB with DVRs and AVRs are shown. Blue arrows indicate water transports, yellow arrows urea transports, thin diffusion, black-rimmed facilitated, hatched with flow. **a **Urea recycling in rat and high concentrators: In the terminal CD a portion of urea is diverted and released by facilitated transport into the VIC of the papilla and taken up by AVRs. Within AVRs urea travels into the VB of the IS where it is transferred by CCE via urea transporter UTA-2 into the SDTL, which descends within the VB. By flow along the nephron route, it reaches the CD in the cortex becoming concentrated by water extraction from the CNT and the entire CD down to the terminal portion and offered for repeated release. **b **Urea recycling in man and low concentrators. The difference to A consists that in B urea is not directly transferred from AVRs into the SDTL, but within the VB urea is first transferred via urea transporter UT-B1 to DVRs that supply the capillary plexus of the IS. From there, in a second step, it is taken up by the SDTL via urea transporter UTA-2. The recycling of urea to the terminal CD is identical. **a and b **Urea trapping: There is no difference in the trapping of urea between high and low concentrators. After uptake of urea into AVRs within the papillary part of the IM urea undergoes CCE with DVRs facilitated by the urea transporter UT-B1 first within the small VBs of the IM, most effective within the large VBs of the IS, decreasing in the OS. A short-circuiting of water from DVRs (via the water channel AQP1) into AVRs increases the effect. This mechanism decreases the loss of urea in the arcuate vein

Two mechanisms underlie the creation and maintenance of the urea gradient: urea recycling and urea trapping (Fig. 12).

### Urea recycling and trapping

 Recycling of urea describes a process that starts with a molecule of urea being reabsorbed from a terminal CD into the VIC of the IM and brings this molecule back into the terminal CD via the tubular route [33, 58]. Urea is taken up by AVRs in the papilla, travels with AVRs into the VBS of the IS where it becomes effectively transferred by CCE into the SDTLs, which in their lower segment express the urea transporter UTA-2 [7]. Via the respective TALs, DCTs, CNTs, CCDs, OMCDs and upper IMCDs, which all are urea impermeable, urea will reach the terminal IMCDs. In anti-diuresis in response to ADH water is reabsorbed from CNTs and CDs resulting in the concentration of urea along the descent achieving the highest concentrations of urea within the terminal CD-segments. There, as described above, a part of urea is reabsorbed into the VIC of the papilla completing the cycle. Perpetuation of this cycle ensures the maintenance of the urea gradient.

Starting with highest concentrations in the papilla urea trapping is ensured by the VB-linked CCE between AVRs and DVRs, facilitated in the latter by the urea transporter UT-B1 [7, 116]. A short-circuiting of water from the AQP1containing DVRs into AVRs [92] increases the effect. Since no urea transporter has been found in any of the thin limb segment in the IM [83] an essential contribution of thin limbs seems unlikely. As urea permeates any cellular membrane, even in the absence of a specific transporter, diffusional exchanges with thin limbs do occur as shown by similar longitudinal concentration profiles as in vasa recta [79].

Within the large VBs of the IS, CCE between AVRs coming up from the IM and SDTLs (bypassing the VIC of the IS, described above) is evident and equally effective with DVRs descending into the IM and, in marginal extent with those supplying the IS; in the OS minimal effects may be added.

These trapping mechanisms minimize the loss of urea into the general circulation.

### The interdependence of the salt and urea gradients in the IM

Urea and salt are the decisive solutes establishing the longitudinal osmotic gradient in the VIC of the IM. Together they provide the osmotic energy needed to extract water from the IMCDs ensuring the final urine concentration. Neither of both gradients can be generated on its own, gradients for salt and urea depend on each other [20].

The enrichment of salt in LDTLs in the IM depends on urea. Together with salt, urea drives water extraction from LDTL segments outlined by epithelium 2a, thus enabling the salt cascade (see above).

The enrichment of urea in CDs along their descent in the IM depends on salt. Together with urea, salt from LDTLs establishes the combined high interstitial osmolality, which drives water extraction from CDs to generate the highest urea concentrations within terminal CDs [20, 99].

Thus, with mutual help, salt and urea reach highest concentrations in the VIC of the papilla creating the basis for the inner medullary solute gradient.

The maintenance of this gradient is ensured by CCE concerning urea, salt and water within the VB-linked CCE system between AVRs and DVRs minimizing the loss of salt and urea into the general circulation. Permanent additions of salt from TALs and of urea from terminal CDs compensate for the inevitable losses.

### Summary of urine concentrating mechanisms in the rat medulla

**Fig. 13:**
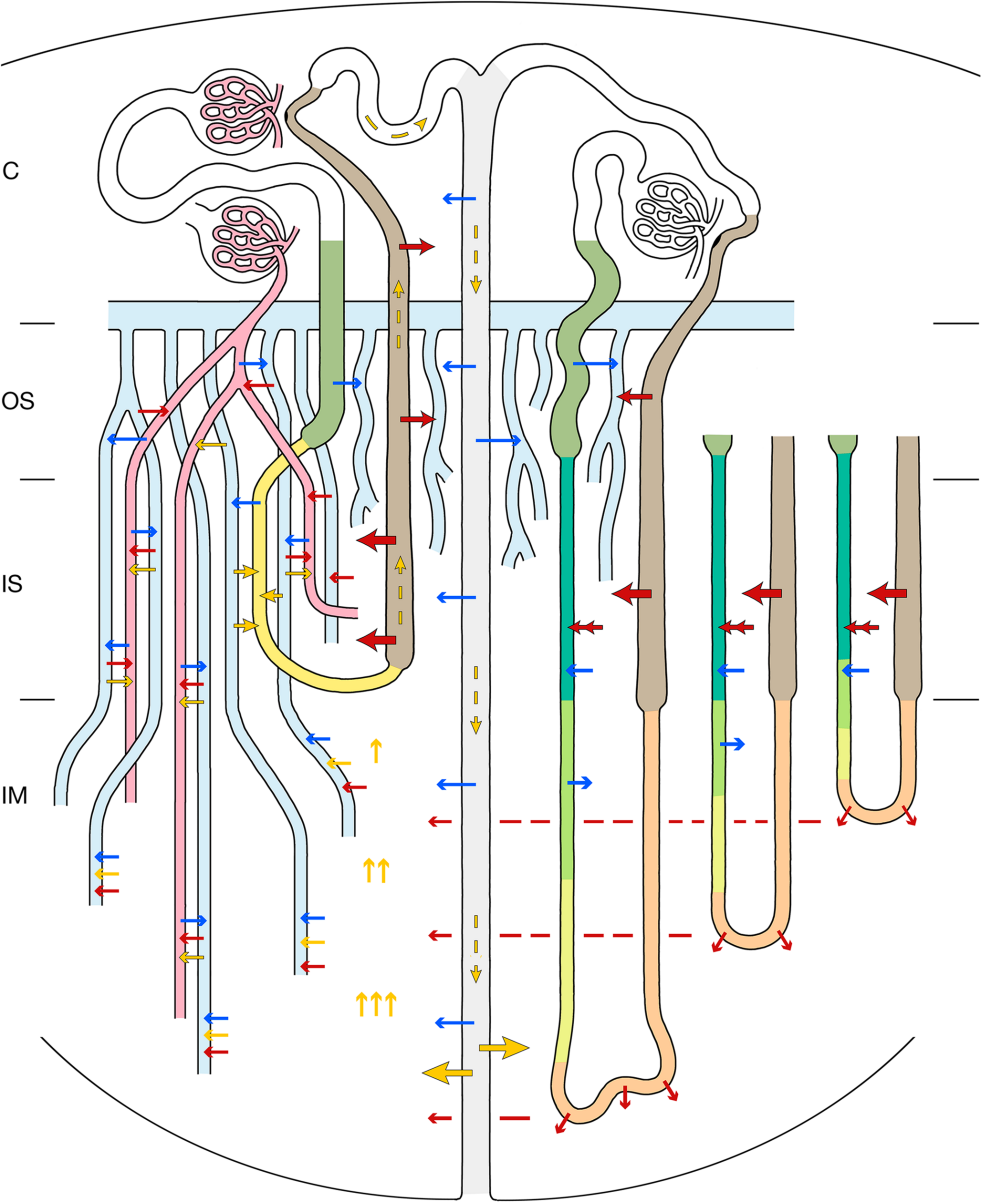
Summary of urine concentrating mechanisms in the rat medulla. Schematic showing the functional interactions in the medulla during antidiuresis. A short-looped nephron, a long-looped nephron with three variations in length, a CD, a VB with DVRs and AVRs and AVRs ascending independently from the VB are shown. Arrows in red, yellow and blue indicate transports of salt, urea and water, respectively. Salt: black-rimmed single headed reabsorption, double headed secretion, thin diffusion. Urea: black-rimmed facilitated transports, thin diffusion, hatched transport by flow. Water: thin, diffusion or transports through channels. OS, IS, and the IM are indicated, not drawn to scale. All white areas outside the VBs represent the VIC comprising the IBR of the OM and IM. For explanations see main text.

The integrated concentrating mechanisms of the urine in the OM and IM is described in direct relation to Fig. 13 subdivided in four steps. First the relevance of the salt gradient, second the relevance of urea gradient, third the combined salt/urea gradient, forth the CCE mechanisms to the maintain the combined gradient.

- First: The salt gradient
  The reabsorption of salt by TALs takes centerstage in the concentrating mechanism. **Step 1**, it loads the AVRs directly ascending from the IS and traversing the OS, which in counter-current arrangement extract water from with PSTs, LDTLs and CDs essentially decreasing the entry of water into the medulla.
  **Step 2**, it creates a salt gradient within the IS which continues water extraction from DTLs and CDs downwards leading in the OM to a basic urine concentration of about twice the plasma concentration.
  **Step 3**, it creates the source for secretion of salt into the LDTLs followed by uptake of a corresponding amount of water providing the fuel for the salt cascade (details see above Fig. 11).
- Second: The urea gradient
  The urea gradient is built up by perpetual releasing a concentrated portion of urea from the terminal CDs and bringing back the diverted portion by urea recycling. This is performed by taking up urea by AVRs in the papilla and transferring it within the VB in the IS by CCE into SDTLs. Via the nephron route urea is brought back into the CD and concentrated by water reabsorption along the entire CDs, warranted by the combined urea and salt gradient.
- Third: The combined urea/salt gradient
  Within the OM the urea gradient plays an insignificant role, since the major part of urea bypasses the VIC of the IS by the LDTLs followed by the usual nephron route. The interstitial gradient is determined predominantly by salt.
  In contrast within the IM urea dominates the combined gradient. Starting with the highest concentrations of urea and salt in the papilla the combined urea/salt gradient gradually decreases within the VIC by uptake of water into AVRs carrying it towards the cortex. The replenishment of urea is warranted by the continuous reabsorption from terminal CDs, that of salt by the bend segments and ATLs along the entire ascent. At the border to the IS all AVRs join the VBs becoming part of the VB-linked vascular CEE system.
- Fourth: maintenance
  The combined medullary urea/salt gradient is maintained by the VB-linked CCE system between AVRs and DVRs, which minimizes the washout of solutes into the general circulation. It starts within the small VBs within the IM, is maximally efficient within the VBs of the IS, strongly decreasing within the small VBs of the OS. Surplus remnants of solutes in AVRs peeling off from the VBs in the OS are used to directly extract water from PSTs and CDs. The final washout through AVRs of salt is continuously balanced by adapted additions from TALs, that of urea by recycling.
  It is worth to mention that ADH regulates all essential steps in the enrichment of salt and urea namely salt reabsorption from TALs, salt secretion into LDTLs, urea concentration along CDs by water reabsorption and urea release from terminal CDs.

## Medullary blood flow

Medullary perfusion is regulated and adapts to the states of diuresis. As described above blood supply of the medulla starts with juxtamedullary EAs. They are sensitive to several mediators including angiotensin II, vasopressin (ADH), endothelin and prostaglandin E2 among others [91] produced in part by the lipid laden inner medullary interstitial cells [32, 68, 82] and CDs.

Compared to the cortex the medullary blood flow is low, clearly lower in AVRs than in DVRs. Moreover, due to CCE within the VBs the oxygen concentrations decrease steeply towards the papilla. Perfusion rates are higher in diuresis than in antidiuresis [113] and high perfusion rates compromise the concentrating process. It is beyond the scope of this review to consider the regulation of medullary perfusion in detail. For the interested reader, the review by Pallone and Cao [91] summarizes the important aspects.

## Urine concentration in other mammalian species including human

A comprehensive overview of the relationship between anatomy and concentrating ability in various species has been published by Bankir and Rouffignac [2], by Sperber [111] and by Schmidt Nielsen [107]. Here we will focus on aspects pertaining to countercurrent processes.

In advance, in the OM the separation of the vasculature into a VB-linked CCE system between DVRs and AVRs and a system of AVRs ascending independently from the VBs essentially decreasing the inflow of water in counter-current arrangement with DTLs and CDs at their entry into the medulla is found in all species.

### Urine concentration in high concentrating species

The list of high urine concentrators considered here comprises in addition to the rat, the mouse [27], the Syrian hamster [1] and the desert rat (Psammomys obesus) [5, 9, 10, 43, 66].

In all these species, the number of short loops clearly surpasses the number of long loops, in the rat 70:30 [56], in the mouse even 82:18 [126], in Psammomys 66:34 [2]. In all of them the SDTLs are incorporated to various degrees into the VBs in the IS, LDTLs descend trough the IBR [58, 64].

Moreover, the LDTLs in the IS are equipped with an outstandingly developed type 2 epithelium with the most prominent elaboration in the longest LDTLs [10, 43].

The mouse kidney has an even higher concentrating ability than the rat (4000 versus 3000 mosmol/L [104, 118]. Specific features concern both types of DTLs. A considerable fraction of SDTLs change their epithelium into the TAL-type already before the bend resulting in the formation of an innermost stripe [65] enforcing the accumulation of salt in the IS. The LDTLs take a tortuous course on their descent through the OM, increasing their length by 27% [126]. The longest LTDLs are equipped in the IS with an extremely elaborated type 2 epithelium.

In the kidney of the hamster (concentrating ability approx.4000 mosmol/L [104] the LDTLs are structured as in rat and mouse with a type 2 epithelium that in some loops is eye-catching thick [1].

In Psammomys (concentrating ability approx. 5000 mosmol/L [2, 107, 118] the blood circulation of OM and IM is most strictly separated, the VBs are fused to giant bundles which include all SDTLs [43]. Most of LDTLs in the IS are equipped with the fully developed type2 epithelium [10], a high proportion of LDTLs form their bends within the tip of the papilla [43].

These specialities in high concentrators are all in line with central roles of salt dragging by LDTLs into the IM and urea recycling via counter-current exchange between sDTLs and AVRs.

### Urine concentration in low concentrating species including man

We consider in this group the human [16, 90, 100, 121], the rabbit [44, 106] and the mountain beaver (aplodontia rufa) [101, 108]. Most detailed knowledge is available from the rabbit kidney, knowledge from the human kidney is incomplete.

The mountain beaver is of interest because it lacks an OM and LDTLs and its urine concentrating ability reaches just two times plasma osmolality and is independent of urea. Thus, the entire concentrating mechanism corresponds to what we describe as basic urine concentrating mechanism in the OM.

The differences of the renal medulla in rabbit and man compared to the high concentrators concern two major features.

First, the SDTLs are not incorporated in the VBs of the IS (Fig. 12b) but descend through the IBR of the IS [64]. Like in high concentrators, AVRs from the IM will traverse the IS within VBs. These AVRs will exchange urea with the DVRs descending into the IM (trapping of urea) but also with DVRs that feed the IBR plexus in the IS [58, 73]. This plexus supplies the SDTLs, which in their lower segment express the urea transporter UTA-2 [7, 8] facilitating the uptake of urea into the SDTLs. The further recycling route back to the terminal CDs is not different from that in high concentrators. Thus, even likely decreased in efficiency, urea recycling as a basic function in the concentrating process is preserved.

Second, the LDTLs start in the IS in man [16] and rabbit [44, 58, 64, 106] with a type 2 epithelium that is clearly less complexly organized than in rat, but in both epithelial stretches are found with a basolateral labyrinth similar to that in the rat. Thus, the secretion of salt may be expected to be preserved but less efficient than in high concentrators.

In the rabbit the concentrating ability is approx.1400mosmol/[104], the ratio between short and long loops is 34:64 [44]. The larger proportion of LDTLs may balance the less complex organisation of the type 2 epithelium.

In humans the concentrating ability is approx.1200mosmol/L [104, 118], the ratio of short to long loops is 85:15 [90, 100]. The short loops include a substantial portion of cortical loops, which form their bends already in the medullary rays of the cortex. Their only contribution to the mechanism of urine concentration may be the dilution of urine in TALs and consequently in the increase of urea concentration along the CNTand CDs.

In conclusion: We consider the concentrating mechanism presented for the rat as basically valid also for the rabbit and the human. The handling of urea is fully consistent, the dragging of salt into the IM seems to work less efficiently.

## Summary

Since more than half a century, countercurrent multiplication (CCM) and countercurrent exchange (CCE) have been in the centre of any discussion about the urinary concentrating process but a role for CCM has never been proven by solid data.

The present proposal in agreement with Anita and Harald Layton [69] abstains from CCM as a basis for the urine concentrating process. Instead, we present a coherent proposal exclusively based on CCE.

Salt reabsorption by the thick ascending limbs of Henle’s loop (TALs) represents the central mechanism in the urine concentrating process.

In the upper parts of the OM, it feeds a system of AVRs extracting water from descending loop limbs and CDs decreasing water entry into the medulla. Ongoing salt reabsorption from TALs into the lower parts of the OM leads to proceeding water extraction from CDs resulting in a twofold increase in urine osmolality at the entry into the IM.

In the IM a longitudinal osmotic gradient underlies the final concentration of the urine. The salt gradient is established by salt secretion into LDTLs combined with water uptake within the IS followed by dragging the salt enriched fluid down into the IM increasing it by a cascade mechanism to highest concentration within the papilla.

This agrees with the view of Robert Berliner and colleagues, who wrote in 1958 [13]: “Instead of acting as a countercurrent multiplier, the loop is viewed as a source of sodium salts and the fact that the loops dip deep into the medulla provides a means of delivering sodium salts deep into the medullary tissue.”

The urea gradient is created along with the concentration of the urine by water extraction from the urea impermeable CDs leading to urea enrichment along their descent toward the papilla. Urea in highest concentrations is accumulated in the terminal CDs. Part of it is diverted and released by facilitated diffusion into the interstitial space resulting in highest urea concentrations in the papilla.

The high concentrations of salt and urea in the papilla are the basis for the formation of a longitudinal osmotic (mainly urea and salt) medullary gradient that is maintained by the VB-linked CCE between AVRs and DVRs in the IM and IS. Within the OS any surplus of solutes in AVRs contributes to the extraction of water from PSTs and CDs. The remnant washout of solutes is balanced by continuous salt addition from TALs and urea by recycling to terminal CDs and release.

ADH controls all essential steps in the proposed model, including salt reabsorption by TALs, salt secretion in LTDLs, water reabsorption from CDs, urea release from terminal CDs and medullary blood flow substantiating the dominant role of ADH in regulating the mechanism of urine concentration.

## Data Availability

No datasets were generated or analysed during the current study.

## Abbreviations

CCM: countercurrent multiplication
CCE: countercurrent exchange
OM: outer medulla
OS: outer stripe
IM: inner medulla
PT: proximal tubule
PST: proximal straight tubule (descending thick limb)
DL: descending limb
DTL: descending thin limb
SDTL: short descending thin limb
LDTL: long descending thin limb
AL: ascending limb
TAL: thick ascending limb
ATL: ascending thin limb
DT: distal tubule
DCT: distal convoluted tubule
CNT: connecting tubule
CD: collecting duct
CCD, OMCD, IMCD: cortical, outer medullary, inner medullary CD
EA: efferent arteriole
DVR: descending vas rectum
AVR: ascending vas rectum
VB: vascular bundle
IBR: inter-bundle region
VIC: vascular-interstitial compartment
